# Cognitive and academic outcomes of large‐for‐gestational‐age babies born at early term: A systematic review and meta‐analysis

**DOI:** 10.1111/aogs.15001

**Published:** 2024-10-30

**Authors:** Xuan Zhao, Alice Poskett, Marie Stracke, Siobhan Quenby, Dieter Wolke

**Affiliations:** ^1^ Department of Psychology, Lifespan Health and Wellbeing Group University of Warwick Coventry UK; ^2^ Warwick Medical School University of Warwick Coventry UK

**Keywords:** academic performance, cognitive, early‐term, gestational age, large‐for‐gestational‐age, macrosomia, neurodevelopment, prematurity

## Abstract

**Introduction:**

Early induction of labor (37^+0^–38^+6^ gestational weeks) in large‐for‐gestational‐age infants may reduce perinatal risks such as shoulder dystocia, but it may also increase the long‐term risks of reduced cognitive abilities. This systematic review aimed to evaluate the cognitive and academic outcomes of large‐for‐gestational‐age children born early term vs full term (combined or independent exposures).

**Material and Methods:**

The protocol was registered in the PROSPERO database under the registration no. CRD42024528626. Five databases were searched from their inception until March 27, 2024, without language restrictions. Studies reporting childhood cognitive or academic outcomes after early term or large‐for‐gestational‐age births were included. Two reviewers independently screened the selected studies. One reviewer extracted the data, and the other double‐checked the data. The risk of bias was assessed using the Newcastle‐Ottawa Quality Assessment Scale. In addition to narrative synthesis, meta‐analyses were conducted where possible.

**Results:**

Of the 2505 identified articles, no study investigated early‐term delivery in large‐for‐gestational‐age babies. Seventy‐six studies involving 11 460 016 children investigated the effects of either early‐term delivery or large‐for‐gestational‐age. Children born at 37 weeks of gestation (standard mean difference, −0.13; 95% confidence interval, −0.21 to −0.05), but not at 38 weeks (standard mean difference, −0.04; 95% confidence interval, −0.08 to 0.002), had lower cognitive scores than those born at 40 weeks. Large‐for‐gestational‐age children had slightly higher cognitive scores than appropriate‐for‐gestational‐age children (standard mean difference, 0.06; 95% confidence interval, 0.01–0.11). Similar results were obtained using the outcomes of either cognitive impairment or academic performance.

**Conclusions:**

No study has investigated the combined effect of early‐term delivery on cognitive scores in large‐for‐gestational‐age babies. Early‐term delivery may have a very small detrimental effect on cognitive scores, whereas being large for gestational age may have a very small benefit. However, evidence from randomized controlled trials or observational studies is required.

## Graphical abstract

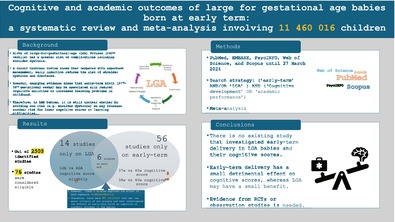

**Abbreviations**
AGA: appropriate‐for‐gestational‐age
CI: confidence interval
GA: gestational age
IQ: Intelligence Quotient
LGA: large‐for‐gestational‐age
OR: odds ratio
SD: standard deviation
SGA: small‐for‐gestational‐age
SMD: standard mean difference

**Key message**

No studies have investigated whether early induction in large‐for‐gestational‐age children impacts their cognitive development. While early term birth is associated with reduced cognition, children born large‐for‐gestational‐age show higher cognitive scores. These effects may balance each other.

## 1 INTRODUCTION

“Large‐for‐gestational‐age” (LGA) generally refers to a birth weight ≥90th percentile of gestational age and, by definition, affects 10% of births.^1^ Birth of an LGA carries a greater risk of complications for both the mother and baby. Shoulder dystocia^2^ of the baby can lead to fractures, brachial plexus injury, hypoxic–ischemic encephalopathy, and death, while risks to the mother include vaginal tears, hemorrhage, and cesarean section.^3^ A Cochrane review showed that compared with expectant management, early induction reduces the risk of shoulder dystocia and fractures.^4^ A large clinical trial of LGA fetuses underway in the United Kingdom is also actively exploring whether early induction of labor starting at 38 weeks will provide perinatal benefits to the fetus and mother.^5^

However, there is evidence showing that early‐term birth (37^+0^–38^+6^ gestational weeks) may be associated with reduced cognitive abilities or increased learning problems in childhood.^6^, ^7^, ^8^, ^9^, ^10^ For example, MacKay et al.^11^ reported a dose‐dependent relationship between gestation at delivery and special educational needs, which is evident throughout gestation. Sixteen to thirty one percent of the population is delivered between 37 and 39 gestational weeks,^12^, ^13^ and a high number of special education needs cases may be attributed to early‐term birth. Even small decreases in cognitive scores can have a marked impact on a child's academic performance at school.^14^ The effects can persist into adulthood,^15^, ^16^ and may affect employment opportunities and income.^17^, ^18^ The latest UK NHS guidance (2019) warns of the increase in special education needs attributed to early‐term births.^19^

This evidence comes from population studies that have not considered indicators of reduced fetal well‐being that require early delivery, including growth restriction, which is an established risk factor for lower cognitive scores from childhood to adulthood.^20^ Therefore, in LGA babies, it is possible that avoiding one risk (shoulder dystocia), which may affect a few, increases another risk (lower cognitive scores or learning difficulties), which would affect many who are subjected to early‐term birth. To date, no systematic reviews have assessed the association between LGA babies born early‐ vs full‐term and their cognitive or academic abilities. Existing systematic reviews that have investigated the effects of only early‐term births on cognitive or academic outcomes do not provide estimates for each specific week of pregnancy before term, which is crucial for planning the timing of induction.^6^, ^7^, ^8^, ^9^, ^10^, ^21^ Empirical evidence is required for pregnant women, their partners, midwives, and obstetricians to make informed decisions on delivery timing.

The present pre‐registered systematic review aimed to systematically review the available evidence on the long‐term cognitive effects of early‐term delivery in large‐for‐gestational‐age babies. However, if there are no existing studies on early‐term LGA babies and their cognitive outcomes, the independent effects of either early‐term birth or LGA should be alternatively reviewed.

## 2 MATERIAL AND METHODS

This systematic review and meta‐analysis was conducted in accordance with the Preferred Reporting Items for Systematic Reviews and Meta‐Analyses (PRISMA) guidelines.^22^ The protocol for this systematic review was registered in the International Prospective Register of Systematic Reviews (PROSPERO) on March 26, 2024 (registration no. CRD42024528626).^23^

### 2.1 Information source and search strategy

X.Z. searched five databases (PubMed, EMBASE, PsycINFO, Web of Science, and Scopus) from their inception to March 27, 2024. Medical Subject Headings (MeSH) and text words were used for the concepts of (1) early‐term delivery, (2) LGA, and (3) cognitive and/or academic outcomes (see Appendix S1). Animal studies, reviews, case reports, editorials, comments, and conference abstracts were excluded. No date or language restrictions were imposed. If the published language was not English, an AI translator (DeepL. SE, Germany) was used for the translation. Additionally, X.Z. performed a manual search based on relevant systematic reviews. All references were imported into EndNote 20 (Clarivate, London, United Kingdom).

### 2.2 Eligibility criteria and selection process

Titles and abstracts were independently screened by two reviewers (X.Z. and A.P. or M.S.) using self‐designed Excel spreadsheets. Potentially eligible studies were then retrieved for full‐text screening. A second eligibility check of the retrieved studies was performed independently by two reviewers (X.Z. and A.P. or M.S.). Any disagreements were fully discussed until a consensus was obtained, and if any uncertainty remained, a third reviewer was consulted (D.W.).

The inclusion criteria were as follows. Studies that evaluated the association between early birth (37^+0^–38^+6^ gestational weeks) and cognitive or academic outcomes were included. Two types of reference groups were used for the comparison of early‐term infants. One study type compared early‐term infants (37–38 weeks) with full‐term infants (39–41 weeks), in which case full‐term infants were the reference group. The second type of study displayed data for each week of gestation (37, 38, 39, 40, and 41 weeks GA) separately, in which case, 40 weeks was used as the reference group to examine 37w vs 40w and 38w vs 40w GA. The second criterion was studies that investigated the cognitive or academic outcomes of being born large‐for‐gestational‐age, defined as a birth weight above the 90th or 80th percentile for gestation defined by whichever growth chart the authors used, either prior to or at delivery. Third, our target sample consisted of healthy children assessed for cognitive scores between 6 months and 18 years of age and healthy individuals assessed for cognitive impairment or low academic performance in all age groups. Studies that targeted populations defined by specific health conditions were excluded. The fourth criterion was the presence of a comparison group, that is, between early‐term and full‐term children (defined as 39^+0^ to 41^+6^ gestational weeks); between children born at 37 or 38 weeks and those born at 40 weeks; and between LGA and appropriate‐for‐gestational‐age (AGA) children (any definition).

### 2.3 Risk of bias assessment

The risk of bias was assessed by two reviewers (X.Z. and M.S.) using the Newcastle–Ottawa Scale, which consists of eight items with a maximum of nine stars. Seven studies judged ≥ 7 stars as low risk, ≥5 as relatively low risk, and ≤4 as high risk. A high‐risk assessment was not a reason for exclusion.

### 2.4 Data extraction

One reviewer (X.Z.) developed standard data extraction forms and modified them according to comments from D.W. and S.Q. Data on author, year, study design, sample size, method of assessing LGA, intervention (induction, spontaneous, cesarean delivery, or not specified), age at follow‐up, cognitive or academic outcomes, and confounders were collected. One reviewer (X.Z.) performed primary data extraction, and a second reviewer (M.S.) checked for accuracy. Any disagreements were resolved by consensus. If the data were insufficient or missing, the corresponding authors of the included papers were contacted via email to provide further details.

Where available, either the mean, standard deviation (SD), total sample size (*N*), or the mean difference, lower/upper limit, and total *N* of the exposed and control groups in the cognitive assessment scores were extracted for the meta‐analysis of continuous variables. For dichotomous variables, either a 2 × 2 table or odds ratio and lower/upper limit were extracted.

All measures of cognitive function were included in this study. When the results were reported as both an overall test score (e.g., Intelligence Quotient; IQ) and a domain‐specific score (e.g., receptive vocabulary delay), the overall score was chosen for data synthesis. When the results were reported as domain‐specific scores within the same study population, the mean score across domain‐specific tests was calculated. Where multiple cognitive or academic outcomes were reported, the one that provided the most reliable information for analysis (e.g. IQ test vs. school grade) was selected. Studies with follow‐ups of at least 6 months were eligible. The oldest age group with the most reliable cognitive assessment was selected when outcomes were measured more than once at different ages. If multiple multivariable models were reported, data from the most confounder‐adjusted model were selected (e.g. adjusted by education and sex vs. adjusted by sex).

Data were extracted based on the following three primary outcomes: Cognitive outcomes were based on cognitive scores (e.g., Bayley Scale of Infant and Toddler Development Mental Developmental Index,^24^, ^25^, ^26^, ^27^, ^28^ with mean: 100; SD: 15 and Wechsler Abbreviated Scale of Intelligence,^29^ with mean: 100; SD: 15) or cognitive impairment (e.g., Wechsler Intelligence Scale for Children‐full scale IQ below average, defined as scores below 85 or one standard deviation below the mean^30^). Academic outcomes were based on low academic performance (e.g., special education needs, defined as children in the Scottish schools 2005 census requiring special education provision, which comprises both children with learning disabilities, such as dyslexia and dyspraxia, and children with physical disabilities that affect learning^11^). For details of the outcome assessments, see Table S4. Data on ADHD diagnoses were extracted as a secondary outcome.

To allow comparability of primary outcomes, harmonization was required using the extracted data: (a) cognitive test T scores, percentiles, or Z scores were converted into intelligence quotient (IQ with mean: 100; SD: 15); (b) outcome directions inconsistent with others (eg, reporting a longer education rather than shorter) were converted to a same‐direction outcome; (c) LGA defined in terms of SD or absolute values was converted to percentiles using the World Health Organization fetal growth calculator (unknown fetal sex).^31^

### 2.5 Data synthesis

Where possible, standardized mean differences or risk ratios were calculated and meta‐analyses were used to describe the findings. Comprehensive Meta‐Analysis (CMA) software (version 4, Professional, Biostat Inc. USA) was used to analyze the data. As the measurement of cognitive and academic outcomes was deemed highly variable, random‐effects models were used in all analyses. The standardized mean difference (SMD) effect size index and 95% confidence interval (CIs) were used for continuous data. When the measurement supplied only dichotomous options, such as cognitive impairment or not, the odds ratio (OR) and 95% confidence interval (CIs) were used. Pooled random effects, 95% prediction intervals, and heterogeneity statistics were calculated for the meta‐analysis, which included at least three studies. Heterogeneity was evaluated using *I* ^2^, Tau,^2^ and *Q* statistics and their *p*‐values. When *I* ^2^ >75%, a series of subgroup meta‐analyses was conducted by splitting the data according to participant characteristics (such as sex and age at follow‐up) or study characteristics (such as outcome measurement scale) to examine the source of heterogeneity. Forest plots were created to provide a graphical overview of individual studies and syntheses.

### 2.6 Publication bias assessment

To assess publication bias, a random‐effects model was used to generate funnel plots for the meta‐analyses. In addition, Duval and Tweedie's trim and fill method was used to estimate the number of missing studies that may exist and the effect that these studies might have had on the outcomes.^32^

### 2.7 Strength of the evidence

The strength of the evidence from all meta‐analyses was independently assessed by two reviewers (X.Z. and M.S.) using the Grading of Recommendations, Assessment, Development, and Evaluation (GRADE) method.^33^ As this review included only observational studies, according to the GRADE guideline, the strength of evidence was initially set as low and was rated down for (1) risk of bias, (2) unexplained heterogeneity of results, (3) indirectness of evidence, (4) imprecision, and (5) publication bias, and the grade was rated up for (1) large effect sizes (relative risk <0.5 or >2, and SMD < −0.25 or >0.25), (2) showing a dose–response relationship, and (3) consideration of the effect of plausible residual confounding (such as parental education level).^34^ The strength of evidence was classified as very low, low, moderate, or high.

## 3 RESULTS

### 3.1 Study selection

**FIGURE 1:**
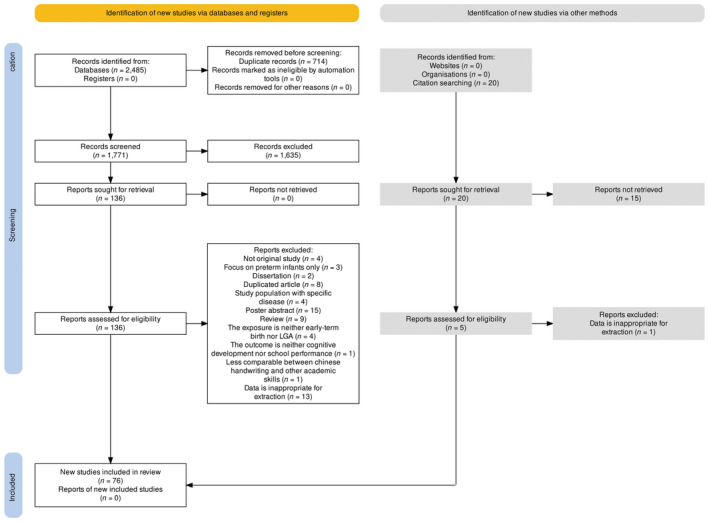
PRISMA flow diagram. LGA, large for gestational age.

Among the 2505 identified citations (2485 from databases and 20 from hand‐searching), no studies met the exact criteria of our initial aim to investigate the effects of early‐term birth on cognitive or academic outcomes in LGA babies. For our alternative aim, a total of 76 articles involving 11 460 016 children were included, as shown in Figure 1.^35^ Fourteen studies investigated only the effect of LGA,^36^, ^37^, ^38^, ^39^, ^40^, ^41^, ^42^, ^43^, ^44^, ^45^, ^46^, ^47^, ^48^, ^49^ 56 studies investigated only the effect of early‐term delivery (37^+0^w‐38^+6^w),^14^, ^24^, ^25^, ^26^, ^27^, ^30^, ^50^, ^51^, ^52^, ^53^, ^54^, ^55^, ^56^, ^57^, ^58^, ^59^, ^60^, ^61^, ^62^, ^63^, ^64^, ^65^, ^66^, ^67^, ^68^, ^69^, ^70^, ^71^, ^72^, ^73^, ^74^, ^75^, ^76^, ^77^, ^78^, ^79^, ^80^, ^81^, ^82^, ^83^, ^84^, ^85^, ^86^, ^87^, ^88^, ^89^, ^90^, ^91^, ^92^, ^93^, ^94^, ^95^, ^96^, ^97^, ^98^, ^99^ and six studies explored the effects of both exposures but reported them independently.^11^, ^29^, ^100^, ^101^, ^102^, ^103^

### 3.2 Study characteristics

The characteristics of the six studies on both the effect of early‐term delivery and large‐for‐gestational‐age on cognitive or academic outcomes are summarized in Table S1, the 14 studies examining the effect of only large‐for‐gestational‐age are shown in Table S2, and details of the 56 studies that investigated only the effects of early‐term delivery are shown in Table S3.

### 3.3 Risk of bias in studies

A summary of the risk assessment results is presented in Table S5. No study was rated as high‐risk (scoring ≤4), 13 studies were rated as relatively low‐risk (scoring≥5),^24^, ^25^, ^27^, ^36^, ^52^, ^53^, ^61^, ^68^, ^69^, ^76^, ^87^, ^104^, ^105^ and all the others were rated as low‐risk.

### 3.4 Early‐term delivery

#### 3.4.1 Cognitive scores

**FIGURE 2:**
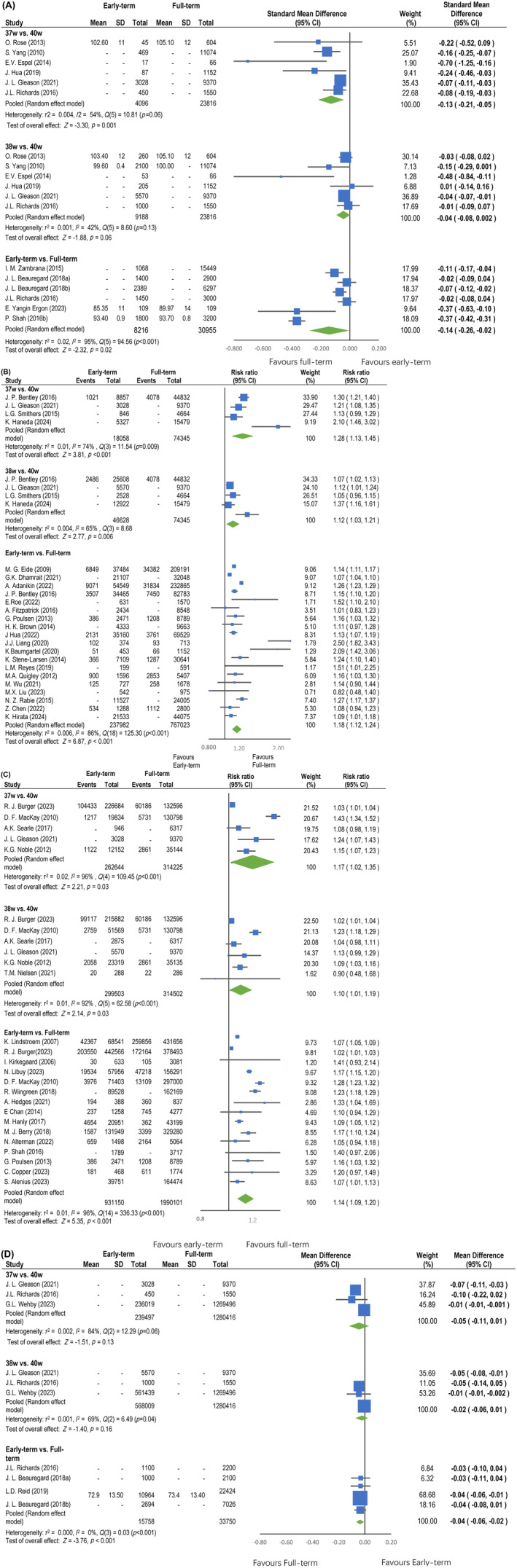
(A) Cognitive scores and early‐term birth. Forest plot for random‐effects meta‐analysis of the association between cognitive scores and early‐term birth. The effect size is expressed as the standardized mean difference (SMD) and 95% confidence interval. The squares represent the estimates, and the size of the squares is proportional to the weight of the individual study in the meta‐analysis. The symbol “‐” indicates that certain values were not available from the study, where alternative values were used to calculate the pooled estimates. CI, confidence interval; SD, standard deviation. (B) Cognitive impairment and early‐term birth. Forest plot for random‐effects meta‐analysis of the association between cognitive impairment and early‐term birth. The effect size is expressed as the odds ratio (OR) and 95% confidence interval. The squares represent the estimates, and their size is proportional to the individual study in the meta‐analysis. The symbol “‐” indicates that certain values were not available from the study, where alternative values were used to calculate the pooled estimates. CI, confidence interval. (C) Low academic performance and early‐term birth. Forest plot for random‐effects meta‐analysis of the association between low academic performance and early‐term birth The effect size is expressed as the odds ratio (OR) and 95% confidence interval. The squares represent the estimates, and their size is proportional to the individual study in the meta‐analysis. The symbol “‐” indicates that certain values were not available from the study, where alternative values were used to calculate the pooled estimates. CI, confidence interval. (D) Average school test scores and early‐term birth. Forest plot for random‐effects meta‐analysis of the association between average school test scores and early‐term birth The effect size is expressed as the standardized mean difference (SMD) and 95% confidence interval. The squares represent the estimates, and their size is proportional to the individual study in the meta‐analysis. The symbol “‐” indicates that certain values were not available from the study, where alternative values were used to calculate the pooled estimates. CI, confidence interval; SD, standard deviation.

Of the studies on the association between early‐term delivery and later cognitive scores, six studies (total *N*: 27912) reported on infants born at 37 weeks gestational age (GA) and cognitive scores in childhood.^24^, ^25^, ^26^, ^27^, ^29^, ^30^ Overall, children born at 37 weeks GA had lower mean cognitive scores than those born at 40 weeks GA (SMD, −0.13; 95% CI, −0.21 to −0.05; *I* ^2^, 54%; low certainty evidence) (Figure 2A). The funnel plot was asymmetric. Using trim and fill, the imputed mean difference and its 95% confidence interval was −0.09 (−0.18, −0.01). The same studies included 33 004 children born at 38 weeks of GA, and the results are shown in Figure 2A. Overall, there was no significant difference between children born at 38 and 40 weeks' GA (SMD, −0.04; 95% CI, −0.08 to 0.002; *I* ^2^, 42%; moderate certainty evidence). Using trim and fill, the imputed mean difference and its 95% confidence interval was −0.04 (−0.09, 0.02). Six studies with a total 39 171 children reported early‐term (37–38 weeks) and cognitive scores (Figure 2A).^27^, ^28^, ^53^, ^84^, ^87^, ^92^, ^93^, ^106^ Overall, early‐term children had lower mean cognitive scores than full‐term children (SMD, −0.14; 95% CI, −0.26 to −0.02; *I* ^2^, 95%; very low certainty evidence). The funnel plot was symmetric. Owing to the high heterogeneity, a subgroup analysis based on age at follow‐up was conducted. The mean differences tended to be smaller in older children (Figure S1).

#### 3.4.2 Cognitive impairment

Cognitive impairment was more common in children born at 37 weeks (92 403 children; OR, 1.28; 95% CI, 1.13–1.45; *I* ^2^, 74%; low certainty evidence) and at 38 weeks (102 973 children; OR, 1.12; 95% CI, 1.03–1.21; *I* ^2^, 65%; moderate certainty evidence) than in those born at 40 weeks (Figure 2B). Cognitive impairment was more common in early‐term than full‐term children (1 005 005 children; OR, 1.18; 95% CI, 1.12–1.24; *I* ^2^, 86%; very low certainty evidence) (Figure 2B).

#### 3.4.3 Low academic performance

Low academic performance was reported more frequently in those born at 37 weeks GA (576 869 children; OR, 1.17; 95% CI, 1.02–1.35; *I* ^2^, 96%; very low certainty evidence; trim and fill, 1.03, 0.91–1.17) and 38 weeks GA (614 005 children; OR, 1.10; 95% CI, 1.01–1.19; *I* ^2^, 92%; low certainty evidence; trim and fill, 1.03, 0.95–1.13) than in those born at 40 weeks (Figure 2C). Low academic performance was more common in early‐term than full‐term children (2 921 251 children; OR, 1.14; 95% CI, 1.09–1.20; *I* ^2^, 96%; very low certainty evidence) (Figure 2C).

#### 3.4.4 Average school test scores

No statistically significant difference was observed in average school test scores between the children born at 37 weeks GA (1 519 913 children; SMD, −0.05; 95% CI, −0.11 to 0.01; *I* ^2^, 84%; low certainty evidence) and 38 weeks GA (1 848 425 children; SMD, −0.02; 95% CI, −0.06 to 0.01; *I* ^2^, 69%; low certainty evidence) compared with those born at 40 weeks (Figure 2D). Average school test scores were slightly lower in early‐term than full‐term children (49 508 children; SMD, −0.04; 95% CI, −0.06 to −0.02; *I* ^2^, 0%; low certainty evidence) (Figure 2D).

#### 3.4.5 Large‐for‐gestational‐age

**FIGURE 3:**
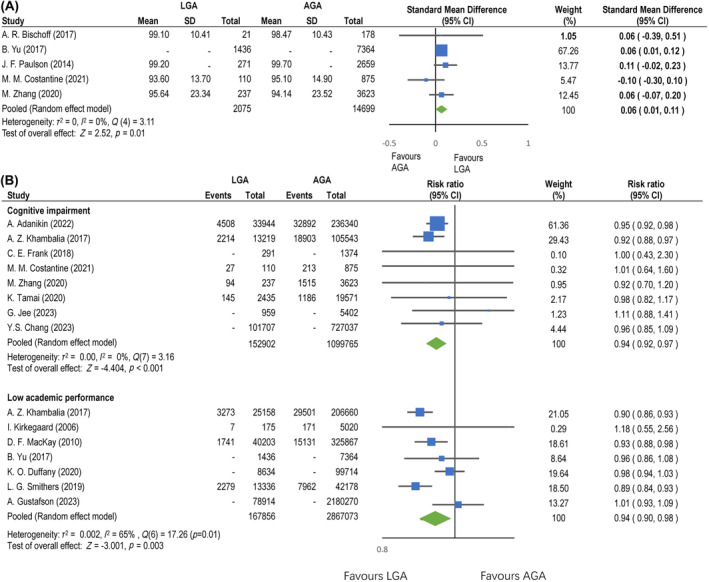
(A) Cognitive scores and large‐for‐gestational‐age. Forest plot for random‐effects meta‐analysis of the association between cognitive scores and large‐for‐gestational‐age. Effect size is expressed as the standardized mean difference (SMD) and 95% confidence interval. The squares represent the estimates, and the size of the squares is proportional to the individual study in the meta‐analysis. The symbol “‐” indicates that certain values were not available from the study, where alternative values were used to calculate the pooled estimates. AGA, appropriate‐for‐gestational age; CI, confidence interval; LGA, large for gestational age; SD, standard deviation. (B) Cognitive impairment/low academic performance and large‐for‐gestational‐age. Forest plot for random‐effects meta‐analysis of the association between cognitive impairment or low academic performance and large‐for‐gestational‐age. The effect size is expressed as the odds ratio (OR) and 95% confidence interval. The squares represent the estimates, and their size is proportional to the individual study in the meta‐analysis. The symbol “‐” indicates that certain values were not available from the study, where alternative values were used to calculate the pooled estimates. AGA, appropriate‐for‐gestational age; CI, confidence interval; LGA, large‐for‐gestational age.

Of 17 (11 plus six) studies investigating the effects of LGA on cognitive or academic outcomes, five studies (*N*: 16774) investigated the association between LGA and later cognitive scores (Figure 3A).^36^, ^37^, ^39^, ^42^, ^43^ Overall, LGA children had higher cognitive scores than AGA children (SMD, 0.06; 95% CI, 0.01–0.11; *I* ^2^, 0%; moderate certainty evidence). Using trim and fill, the imputed mean difference and 95% confidence interval was 0.05 (0.01, 0.10). Eight studies (*N*: 1252667) evaluated the association between LGA and cognitive impairment^38^, ^42^, ^43^, ^45^, ^46^, ^47^, ^49^, ^100^ while another seven studies (*N*: 3034929) used low academic performance^11^, ^37^, ^40^, ^41^, ^46^, ^48^, ^102^ as an outcome measure. The results are presented in Figure 3B. Overall, cognitive impairment was less common in LGA children than AGA children (OR, 0.94; 95% CI, 0.92–0.97; *I* ^2^, 0%; moderate certainty evidence), as was low academic performance (OR, 0.94; 95% CI, 0.90–0.98; *I* ^2^, 65%; moderate certainty evidence). For studies on cognitive impairment, the imputed odds ratio and 95% confidence interval using trim and fill was 0.94 (0.91, 0.97). For low academic performance, the imputed odds ratio and 95% confidence interval was 0.90 (0.86, 0.95) using trim and fill.

## 4 DISCUSSION

This systematic review detected no study before March 27, 2024, that investigated cognitive scores, cognitive impairment, or low academic performance in early‐term births of large‐for‐gestational‐age babies. Existing studies have only independently investigated the effects of LGA against AGA or early‐term births (37–38 weeks GA) compared with full‐term births (39–41 weeks GA) on cognitive outcomes. Alternatively, they used one of these exposures as a confounder to adjust for this factor in association with the cognitive outcomes. Early‐term children born at 37 weeks but not at 38 weeks were found to have slightly lower cognitive scores, a slightly increased risk of cognitive impairment, and lower academic performance than children born at full term. Within the early‐term birth group, those born at 37 weeks’ GA tended to have a slightly higher risk than those born at 38 weeks’ GA, compared with those born full‐term. This is consistent with the well‐recognized dose–response relationship between GA and cognitive outcomes throughout the gestational age range. LGA children had slightly higher cognitive scores, less frequent cognitive impairment or lower academic performance in childhood than AGA children. This evidence has moderate certainty.

The results slightly favored full‐term vs. early‐term delivery for cognitive or academic outcomes. According to Cohen's D of means, a two‐point difference in intelligence quotient (IQ) refers to a very small effect size^107^ (e.g., −0.14 standard difference in means × 15 points = −2.10 IQ difference). When early deliveries were examined separately by week of gestation, only a very small clinically significant difference in IQ was found between children born at 37 weeks and those born at 40 weeks, while no significant difference was found between those born at 38 weeks and those born full term. Considering that 16%–31% of fetuses are born early, the effect of early term on the overall population IQ may be a reduction of between 0.4 to 0.7 IQ points maximum, a very small effect.

The comparison of LGA vs AGA births slightly favored those born LGA, but the difference was not clinically significant. No study was published before March 27, 2024, that considered the combined effect of early‐term birth and large‐for‐gestational‐age on cognitive and academic outcomes. Thus, it cannot be ruled out that the small effect of early‐term birth may be partly due to confounding by SGA fetuses being more frequently delivered early. SGA is a known factor associated with lower cognitive outcomes.^108^ Alternatively, gestational age and weight percentile at birth have additive effects on cognitive development; therefore, in early born LGA babies, the two effects may offset each other to some extent.^109^

Several systematic reviews^6^, ^7^, ^8^, ^9^, ^10^ that have evaluated the relationship between early delivery and cognitive or academic outcomes are consistent with the conclusions of our review. However, these systematic reviews only conducted a qualitative synthesis of the included literature, with no quantitative meta‐analysis. In terms of fetal growth, although a systematic review and meta‐analysis of good quality showed that SGA is detrimental to cognitive development,^108^ we found no extant systematic reviews synthesizing evidence of cognitive development or academic performance in children with LGA.

The present systematic review provides the best available evidence of cognitive and academic outcomes in early‐term LGA babies and of early‐term birth or LGA on cognitive outcomes. The strength of this review was that a pre‐registered protocol was followed to search for articles without time or language constraints. Population sizes were large across several countries, and follow‐ups were carried out during childhood.

One limitation of this study was the high heterogeneity of measurements of IQ and in the definition of cognitive impairment and low academic performance. The included studies used variable metrics to report results, and some studies with missing metrics (e.g., SD of the mean) had to be excluded from the meta‐analyses. Furthermore, different definitions have been used for LGA or full‐term infants across studies. For example, although most studies used the 90th percentile as the definition of large‐for‐gestational‐age, several studies used the 80th^41^ or 85th^36^, ^43^ percentile as the definition. Although most studies used the 10th–90th percentile as the definition of AGA, some studies used other reference groups, for example, 20th–79th^41^ or 85th–90th percentile.^44^ Although all fall within the official definition of the 10th–90th percentile, the use of different reference groups is likely to alter the effect sizes of the comparisons. Similarly, Libuy's (2023)^72^ use of births at 39^+0^–40^+6^ weeks (rather than 39^+0^–41^+6^ weeks) as a full‐term reference group also poses a risk of bias in data synthesis. In addition, only a few studies were stratified according to sex; therefore, the effect of sex could not be evaluated. Moreover, although we excluded studies based on populations with specific health conditions, such as maternal diabetes and congenital anomalies, a significant proportion of the included studies did not exclude specific populations (eg, Mackay 2010^11^) or excluded some and not others (eg, Adanikin 2022^100^). Therefore, we could not evaluate the effects of specific health conditions. Finally, only a few studies reported crude results and data adjusted for covariates. Sensitivity analysis using crude and adjusted results based on available data revealed no substantial differences (Table S6).

There is a paucity of studies stratifying children born in the same gestational week according to their birthweight percentile (or vice versa). Analyses by gestational age or LGA separately cannot determine whether these effects are additive or moderate to each other. First, observational studies are required to investigate the effects of LGA or relative birthweight at each gestational week on cognitive and academic outcomes. A new study published in March 2023 utilized four cohort studies (*N* = 30 643)^109^ and reported that relative birthweight (birthweight percentile per gestation) and gestational week independently affect cognitive scores in childhood, and are thus additive. IQ linearly increased by 4.2 points as birthweight percentiles increased from the first to the 69th percentile before completely plateauing (ie no more IQ gain at larger birthweight). After 32 weeks of gestation, each GA week gained was associated with a 0.3 IQ increase, similar to previous studies.^110^ Future studies are needed to report the relative birthweight per gestational week and its effect on cognitive outcomes for obstetric decision‐making. Second, a randomized clinical trial would be desirable comparing the effect of early‐term induction of LGA vs. expectant delivery and the effect of this intervention to reduce shoulder dystocia on the long‐term cognitive and academic outcomes of the child. Considering the small effect sizes suggested, a large number of participants is required.

## 5 CONCLUSION

Based on the current evidence and considering all provisions outlined above, we conclude that birth at 38 weeks for LGA babies is unlikely to reduce cognitive outcomes to a clinically significant degree at the population level. We advise caution regarding early induction at 37 weeks, which may cause a small reduction in IQ in a large number of infants. Nevertheless, the reduction in cognitive outcomes was clinically very small, and the intervention may prevent a potentially catastrophic perinatal event in a small number of babies. For LGA children in particular, more research is needed to confirm whether the slight advantage of being larger is significant when compared with the risks that LGA babies face going to 40 or 41 weeks gestation (e.g., hypoxic–ischemic encephalopathy). These studies will contribute greatly to helping obstetricians and parents weigh the advantages and disadvantages and make the best possible decisions about the timing of labor.

## AUTHOR CONTRIBUTIONS

**Xuan Zhao**: designed and conceived the study, designed the methodology, undertook a literature search, conducted the study screening, performed the data extraction and risk assessment, performed statistical analyses and designed the tables, figures, and online supplements and prepared the initial drafts of the manuscript. **Siobhan Quenby**: designed and conceived the study and gave input to original draft. **Dieter Wolke**: designed and conceived the study, designed the methodology, checked study screening and prepared the initial drafts of the manuscript. **Alice Poskett**: conducted the study screening and gave input to original draft. **Marie Stracke**: conducted the study screening, performed the data extraction and risk assessment, and gave input to original draft. All the authors contributed to the final version of this manuscript.

## FUNDING INFORMATION

Xuan Zhao is supported by the Chancellor's International Scholarship of the University of Warwick and the BB2UP PhD Fellowship (supported through a legacy gift from University of Warwick alumnus Jack Straw (BSc Mathematics and Economics, 1969‐72)); Prof. Quenby, Prof. Wolke, Alice Poskett, and Marie Stracke are also supported by the BB2UP Grant. Prof. Wolke is additionally supported by a UKRI Frontier Research grant (EP/X023206/1) under the UK government's funding guarantee for ERC‐AdG grants.

## CONFLICT OF INTEREST STATEMENT

The authors declare no conflicts of interest.

## Supporting information


Appendix S1.

Appendix S2.

Appendix S3.

Figure S1.

Figure S2.

Table S1.

Table S2.

Table S3.

Table S4.

Table S5.

Table S6.

Table S7.
