# Supplementary material for: Cognitive and academic outcomes of large‐for‐gestational‐age babies born at early term: A systematic review and meta‐analysis
Source: Acta Obstet Gynecol Scand. 2024 Oct 30;104(2):288–301. doi: 10.1111/aogs.15001 (PMC11782071; doi:10.1111/aogs.15001)
Supplement: Supplementary file 1 — Appendix S1. [file AOGS-104-288-s011.docx]

**PubMed searching strategy (amended)**

1. ((((“macrosomi*”[Title/Abstract]) OR (“large for gestational age”[Title/Abstract]) OR (“LGA”[Title/Abstract]) OR (“large-for-gestational age”[Title/Abstract]) OR (“large bab*”[Title/Abstract]) OR (“big bab*”[Title/Abstract]) OR (“large fetus*”[Title/Abstract]) OR (“big fetus*”[Title/Abstract]) OR (“large foetus*”[Title/Abstract]) OR (“big foetus*”[Title/Abstract]) NOT (“animals”[MeSH Terms])

AND/OR

2. ((((((“early term”[Title/Abstract]) OR (37 to 38 weeks[Title/Abstract]) OR (37 to 38 gestational weeks[Title/Abstract]) OR (“late preterm”[Title/Abstract]) OR (“marginal preterm”[Title/Abstract]) NOT (“animals”[MeSH Terms])

AND

3. (((((((((((((((((“cognition”[MeSH Terms])) OR (“cognitive”[Title/Abstract])) OR (“neurodevelopmen*”[Title/Abstract])) OR (“language development”[Title/Abstract])) OR (“non-verbal development”[Title/Abstract])) OR (“school performance”[Title/Abstract])) OR (“math*”[Title/Abstract])) OR (“academic achievement*”[Title/Abstract])) OR (“reading”[Title/Abstract])) OR (“writing”[Title/Abstract])) OR (“vocabulary”[Title/Abstract])) OR (“intelligen*”[Title/Abstract])) OR (“special education needs”[Title/Abstract])

4. 1 AND/OR 2 AND 3

Web of Science search strategy

**(TS=(macrosomi*) OR TS=("large for gestational age") OR TS=("LGA") OR TS=("large bab*") OR TS=("big bab*") OR TS=("large fetus*") OR TS=("big fetus*") OR TS=("large foetus*") OR TS=("big foetus*")) NOT TS=(animals)**

**TS=("early term") OR TS=(37 to 38 weeks) OR TS=("37 to 38 gestational weeks") OR TS=("late preterm") OR TS=("marginal preterm") NOT TS=("animals")**

**TS=("cognition") OR TS=("cognitive") OR TS=("neurodevelopmen*") OR TS=("language development") OR TS=("non-verbal development") OR TS=("school performance") OR TS=("math*") OR TS=("academic achievement*") OR TS=("reading") OR TS=("writing") OR TS=("vocabulary") OR TS=("intelligen*") OR TS=("special education needs")**

**#2 AND #1 AND #3**

**(#2 OR #1) AND #3**

Scopus Search Strategy

1. TITLE-ABS-KEY ( macrosomi* ) OR TITLE-ABS-KEY ( "large for gestational age" ) OR TITLE-ABS-KEY ( "LGA" ) OR TITLE-ABS-KEY ( "large bab*" ) OR TITLE-ABS-KEY ( "big bab*" ) OR TITLE-ABS-KEY ( "large fetus*" ) OR TITLE-ABS-KEY ( "big fetus*" ) OR TITLE-ABS-KEY ( "large foetus*" ) OR TITLE-ABS-KEY ( "big foetus*" ) NOT TITLE-ABS-KEY ( animals )
2. TITLE-ABS-KEY ( "early term" ) OR TITLE-ABS-KEY ( 37 to 38 weeks ) OR TITLE-ABS-KEY ( "37 to 38 gestational weeks" ) OR TITLE-ABS-KEY ( "late preterm" ) OR TITLE-ABS-KEY ( "marginal preterm" ) NOT TITLE-ABS-KEY ( animals )
3. TITLE-ABS-KEY ( "cognition" ) OR TITLE-ABS-KEY ( "cognitive" ) OR TITLE-ABS-KEY ( "neurodevelopmen*" ) OR TITLE-ABS-KEY ( "language development" ) OR TITLE-ABS-KEY ( "non-verbal development" ) OR TITLE-ABS-KEY ( "school performance" ) OR TITLE-ABS-KEY ( "math*" ) OR TITLE-ABS-KEY ( "academic achievement*" ) OR TITLE-ABS-KEY ( "reading" ) OR TITLE-ABS-KEY ( "writing" ) OR TITLE-ABS-KEY ( "vocabulary" ) OR TITLE-ABS-KEY ( "intelligen*" ) OR TITLE-ABS-KEY ( "special education needs" )
4. #1 AND #2 AND #3
5. (#1 OR #2) AND #3

Embase & psychoINFO search strategy

1. ((macrosomi* or "large for gestational age" or "LGA" or "large bab*" or "big bab*" or "large fetus*" or "big fetus*" or "large foetus*" or "big foetus*") not "animals").mp.
2. (("early term" or "37 to 38 weeks" or "37 to 38 gestational weeks" or "late preterm" or "marginal preterm") not "animals").mp.
3. ("cognition" or "cognitive" or "neurodevelopmen*" or "language development" or "non-verbal development" or "school performance" or "math*" or "academic achievement*" or "reading" or "writing" or "vocabulary" or "intelligen*" or "special education needs").mp.
4. 1 and 2 and 3
5. (1 or 2) and 3
