# Supplementary figures and images for: Cognitive and academic outcomes of large‐for‐gestational‐age babies born at early term: A systematic review and meta‐analysis
Source: Acta Obstet Gynecol Scand. 2024 Oct 30;104(2):288–301. doi: 10.1111/aogs.15001 (PMC11782071; doi:10.1111/aogs.15001)

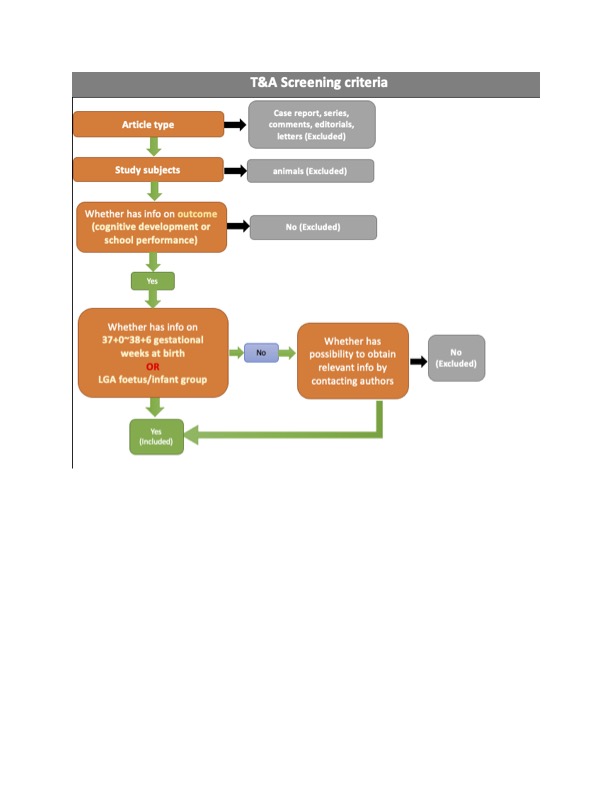

Supplement: Supplementary file 2 — Appendix S2. [file AOGS-104-288-s006.jpg]

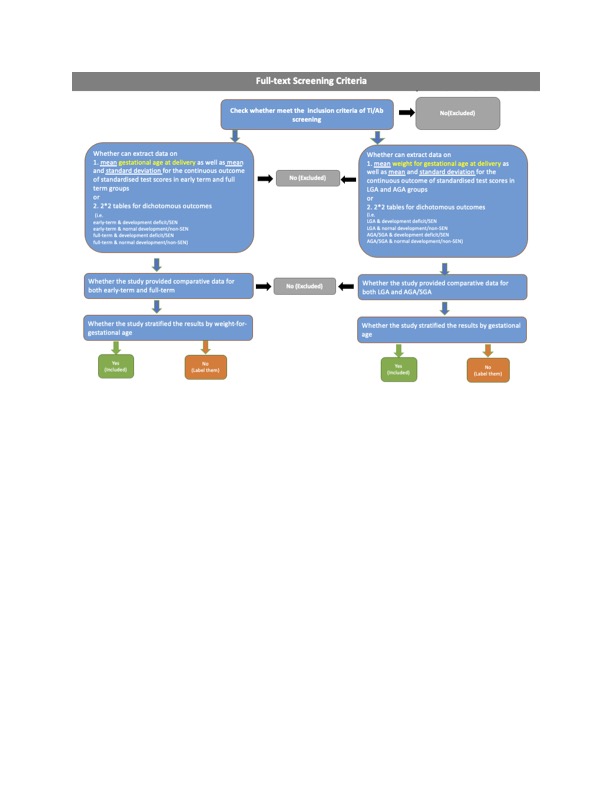

Supplement: Supplementary file 3 — Appendix S3. [file AOGS-104-288-s005.jpg]

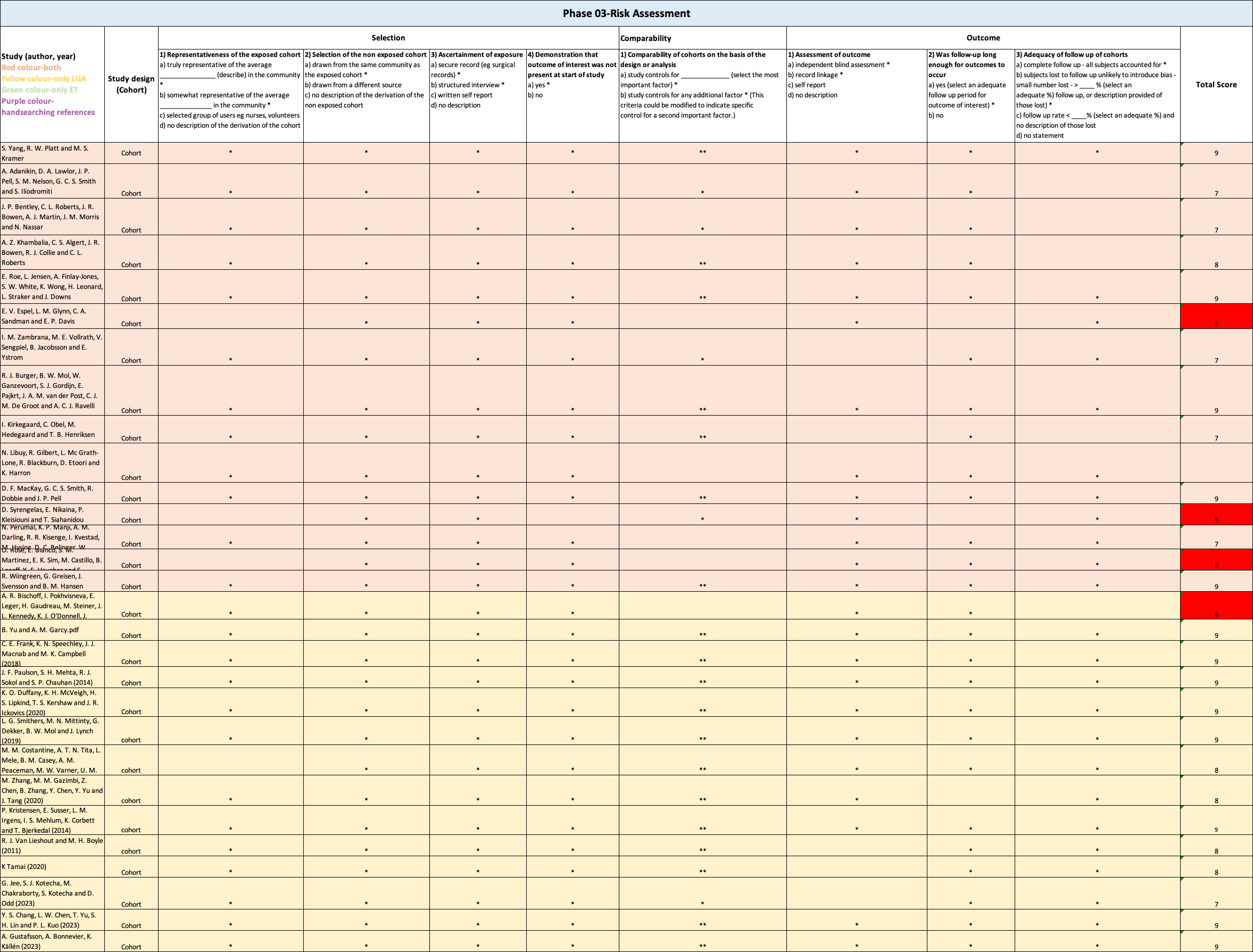

Supplement: Supplementary file 10 — Table S5. [file AOGS-104-288-s003.zip › Table S5_1.docx]
