# Supplementary material for: Cognitive and academic outcomes of large‐for‐gestational‐age babies born at early term: A systematic review and meta‐analysis
Source: Acta Obstet Gynecol Scand. 2024 Oct 30;104(2):288–301. doi: 10.1111/aogs.15001 (PMC11782071; doi:10.1111/aogs.15001)
Supplement: Supplementary file 6 — Table S1. [file AOGS-104-288-s010.docx]

Table S1 Characteristics of studies investigating the effects of both early-term delivery and large for gestational age on cognitive /academic outcomes

| **Study (Year)** | **Study design** | **Country** | **Exposures** | **Early-term /Full-term sample size** | **LGA/AGA sample size** | **Investigated effects of early-term and LGA in combination or independently** | **Follow-up years** | **Outcomes** | **Outcomes ascertainment** |
| --- | --- | --- | --- | --- | --- | --- | --- | --- | --- |
| A. Adanikin et al. (2022)(91) | Cohort study | United Kingdom | Early-term delivery (defined as 37+0~38+6 weeks) &  LGA (defined as >90^th^ percentile) | 54549  /232865 | 33944  /236340 | Independently | 2-3.5 years | Cognitive impairment | The Ages and Stages Questionnaire (ASQ-3)  Child Health Surveillance Programme–Preschool (CHSP-PS) |
| M. Sucksdorff et al. (2015)(92) | Registry | Finland | Early-term delivery (defined as 37+0~38+6 weeks) &  LGA (defined as >1.5 SD) | 8839  /35105 | 3663  /17901 | Independently | 3-19 years | ADHD | ICD 10th |
| S. Yang et al. (2010)(29) | Cohort study | The Republic of Belarus | 37/38 weeks delivery  &  LGA (defined as >90^th^ percentile) | 469/11074  (37 weeks)  2100/11074  (38 weeks) | N/A | Independently | 6.5 years | Cognitive score | Wechsler Abbreviated Scales of Intelligence (WASI) |
| I. Kirkegaard et al. (2006)(93) | Cohort study | Denmark | Early-term delivery (defined as 37+0~38+6 weeks) &  LGA (defined as >=4500g) | 633/3081 | 175/1942 | Independently | 9-11 years | Low academic performance | Reading/spelling/arithmetic difficulties by Parents' and teachers' report |
| D. F. Mackay et al. (2010)(11) | Cohort study | United Kingdom | 37/38 weeks delivery  &  LGA (defined as >90^th^ percentile) | 19834/130798  (37/40 weeks)  51569/130798  (38/40 weeks) | 40203  /325867 | Independently | 4-19 years | Low academic performance | Children who have been provided special education support; data extracted from Scotland School census |
| M.G. Eide et al. (2007)(94) | Registry | Norway | Early-term delivery (defined as 37+0~38+6 weeks) &  LGA (defined as > 3 SD) | 37484/209191 | 12461  /52484 | Independently | 18 years | Cognitive impairment | <=3 out of 9 measured by a 53-min group intelligence test (developed in 1953 in Norway) |
