# Supplementary material for: Cognitive and academic outcomes of large‐for‐gestational‐age babies born at early term: A systematic review and meta‐analysis
Source: Acta Obstet Gynecol Scand. 2024 Oct 30;104(2):288–301. doi: 10.1111/aogs.15001 (PMC11782071; doi:10.1111/aogs.15001)
Supplement: Supplementary file 7 — Table S2. [file AOGS-104-288-s009.docx]

Table S2 Characteristics of studies investigating the effects of only large for gestational age on cognitive /academic outcomes

| **Study (Year)** | **Country** | **Study Design** | **LGA/AGA total sample size** | **Method of assessing weight percentile** | **Definition of LGA** | **Age at follow-up** | **Outcomes** | **Outcomes ascertainment** |
| --- | --- | --- | --- | --- | --- | --- | --- | --- |
| A. R. Bischoff (2017)(35) | Canada | Cohort study | 21/  178 | Birth weight ratio (BWR) (the ratio between the observed birth weight and the sex-specific mean birth weight for each gestational age for the local population) | >BWR 1.15 (harmonized as >84^th^ centile) | 3 years | Cognitive score | Bayley Scales of Infant and Toddler Development II: Mental Developmental Index (MDI) |
| B. Yu (2017)(36) | Sweden | Cohort Study | 1436/  7364 | New Dutch reference curves for birthweight by gestational | >90th centile | 13 years; 48 years | Cognitive score & low academic performance | Cognitive score was assessed by verbal, spatial, and numerical ability tests (each with a 40-point scale); Low academic performance was based on attained lower education defined as lower level than post-secondary education |
| C. E. Frank (2018)(37) | Canada | Cohort study | 311/  1374 | Sex-specific Canadian standards | >90^th^ centile | 4-5 years | Cognitive score | Assessed using the Revised Peabody Picture Vocabulary Test (PPVT-R) |
| J. F. Paulson (2014)(100) | United States | Cohort study | 271/  2659 | Gender-specific US-standard birth weights for gestational age reference values(105) | >90^th^ centile | 9 months, and 2, 3.5, and 5.5 years. | Cognitive score | Assessed by the Mental Developmental Subscale of the Bayley Scales of Infant Development (BSID) (9 months); Assessed by cognitive tests developed by the research team (4-5 years) |
| K. O. Duffany (2020)(39) | United States | Cohort study | 8634/  99714 | Gender-specific US-standard birth weights for gestational age reference values(105) | >90th centile | 8-9years (3rd grade) | Low academic performance | Did not meet the mathematic or English proficiency in a national education assessment in the US |
| L. G. Smithers (2019)(40) | Australia | Registry study | 13368/  42256 | Z scores of birth weight for gestational age (BWGA) were calculated using Australian norms | >80th centile | 8 years | Low academic performance | The National Assessment Program - Literacy and Numeracy (NAPLAN), consists of assessments of reading, writing, spelling, grammar, and numeracy skills for third-grade students. Scores are categorized as above or not above the national minimum standards. |
| M. M. Costantine (2022)(41) | United States | Cohort study | 110/875  (Alexander)  82/777 (National Fetal Growth) | 1. ethnicity- and sex-specific population nomogram (Alexander)(106)  2. National Fetal Growth (NFG)(107) | >90th centile | 5 years | Cognitive score | Wechsler Preschool and Primary Scale of Intelligence – Third  Edition (WPPSI-III) Full Scale test. |
| M. Zhang (2020)(42) | China | Cohort study | 237/3623 | Absolute birthweight | >4000g  (Harmonized as >80^th^ centile) | 1-6 months | Cognitive impairment | <85 on the Gesell Development Scale |
| P. Kristensen (2014)(43) | Norway | Registry study | N/A | Absolute birthweight | >4500g  (Harmonised as >97^th^ centile) | 18-19 years | Cognitive score | Intellectual Performance score from the Norwegian Armed Forces Personnel Database (transformed to an IQ scale) |
| A. Z. Khambalia (2017)(45) | Australia | Registry study | 49439/ 400418 | N/A | >90th centile | 4-7years 3years | Cognitive impairment | <10^th^ centile on >=1 Australian Early Development Index (AEDI) domain |
| K. Tamai (2020)(44) | Japan | Cohort study | 3724/  29725 | The study used the SD of birth weight for gestational age from the Committee for Newborns of the Japanese Pediatric Society to classify the participants. | >=1.28 SD  (Equals 90^th^ centile) | 2.5 years  5.5 years | Cognitive impairment | Unable to reach age-appropriate developmental milestones (unable to listen carefully, unable to focus on one task, unable to remain patient, unable to express emotions, unable to act in group, unable to keep promise) from parents’ reports |
| G. Jee (2023) | Wales, UK | Cross-sectional study | 959/  5402 | Birthweight centile were calculated using the LMS Growth program. | >90^th^ centile | Not specified | Cognitive impairment | Parent-reported neurodevelopment outcomes (for example speech problems) through questionnaires |
| Y.S. Chang (2023) | Taiwan | Registry study | 101707/  727037 | Birthweight centile was calculated by ranking the index case in the study cohort of the same sex and same gestational age. | >90^th^ centile | Not specified | Cognitive impairment | Intellectual disability determined by the ICD-9-CM codes 317-319 |
| A. Gustafsson (2024) | Sweden | Registrystudy | 78914/  2180270 | Birthweight centile was calculated according to the Swedish growth standard curve | >2 SD | 16 years | Low academic performance | Special education needs defined by children who left mainstream school at 16 years |
