# Supplementary material for: Cognitive and academic outcomes of large‐for‐gestational‐age babies born at early term: A systematic review and meta‐analysis
Source: Acta Obstet Gynecol Scand. 2024 Oct 30;104(2):288–301. doi: 10.1111/aogs.15001 (PMC11782071; doi:10.1111/aogs.15001)
Supplement: Supplementary file 8 — Table S3. [file AOGS-104-288-s008.docx]

Table S3 Characteristics of studies investigating the effects of only early-term delivery on cognitive /academic outcomes

| **Study (Year)** | **Study design** | **Country** | **Early-term /Full-term sample size** | **Follow-up years** | **Outcomes** | **Outcomes ascertainment** |
| --- | --- | --- | --- | --- | --- | --- |
| A. Fitzpatrick (2016) | Cohort | United Kingdom | 2434/ 8548 | 3, 5, 7 and 11 years | Cognitive impairment | British Ability Scales Edition II Verbal Similarities test, Cambridge Neuropsychological Test Automated Battery Spatial Working Memory test. |
| A. Hedges  (2021) | Cohort | United States of America | 388/ 837 | 1, 3, 5 and 9 years | Academic Performance | Interviews with mothers, Teacher's reports incl. Cognitive Problems/Inattention  Subscale of the Conners Teacher Rating Scale–Revised Short Form (CTRS-RSF) |
| A. K. Searle,  (2017) | Registry | Australia | 3821/ 6317 | 8 years | Academic performance | National Assessment Program—Literacy and Numeracy (NAPLAN) |
| A. S. Hodel (2016) | Cohort | United States of America | - | 4 years | Cognitive impairment | modified delay discounting paradigm, Maudsley’s Index of Childhood Delay Aversion (MIDA), measure of short-term spatial memory and spatial working memory, standard computerized go/ no-go paradigm, WPPSI-III, Behaviour Rating Inventory of Executive Function |
| E. Chan (2014) | Cohort | United Kingdom | 1258/ 4277 | 9 months, 3, 5 and 7 years | Academic performance | Key Stage 1 |
| G. Dueker (2016) | Cohort | United States of America | - | 8, 12, 20 or 24 months | Cognitive impairment | Ages and Stages Questionnaire (ASQ) |
| G. K. Dhamrait  (2021) | Registry | Australia | 21107/ 32048 | 4-6 years | Cognitive impairment | AEDC, originally the Australian Early Development Index (AEDI) |
| G. Lingasubramanian (2022) | Cohort | United States of America | 400/ 1022 | 9 years | ADHD | Conners Teacher Rating Scale–Revised Short Form (CTRS-RSF) |
| G. Poulsen (2013) | Cohort | United Kingdom | 3655/ 12540 | 3,5 and 7 years | Cognitive impairment | Selected subscales from the British Ability Scale, second edition (BAS II), the Bracken School Readiness Assessment (BSRA) and a mathematics assessment, Numeracy Skills |
| O. Gale-Grant, (2021) | Cohort | United Kingdom | - | 18 months | Cognitive impairment | Bayley Scales of Infant and Toddler Development, Third Edition (Bayley-III) |
| H. K. Brown, (2014) | Cohort | Canada | 3502/ 7810 | 2-5 years | Cognitive impairment | Motor and Social Development Scale, Peabody Picture Vocabulary Test–Revised (PPVT-R) |
| H. S. Lipkind, (2012) | Cohort | United States of America | - | 8-9 years | Academic performance | standardized scores from DOE-administered third-grade English Language Arts (ELA) and math tests. |
| J. Hua, (2022) | Cohort | China | 35160/ 69529 | 3-5 years | Cognitive impairment | Ages and Stages Questionnaire 3 (ASQ3) |
| J. Hua (2019) | Cohort | China | 292/ 1152 | 16 days to 48 months | Cognitive impairment | The Bayley Scale of Infant and Toddler Development, Third Edition (BSID-III) |
| J. J. Liang, (2020) | Cohort | China | 374/ 713 | 1 year | Cognitive impairment | Gesell Developmental Scale (GDS) |
| J. L. Beauregard, (2018a) | Cohort | United States of America | 1600/ 3350 | 9 months, 2 and 5-6 years | Cognitive impairment, academic performance | Bayley Short Form–Research Edition (BSF-R), ECLS-B–designed assessments of knowledge and skills in reading and mathematics |
| J. L. Beauregard, (2018b) | Cohort | United Kingdom | 3368/ 8707 | 3, 5 and 7 years | Cognitive impairment | Bracken School Readiness Assessment Revised (BSRA-R) and British Ability Scales II (BAS II), National Foundation for Education Research (NFER) number skills |
| J. L. Gleason, (2021) | Cohort | United States of America | 8598/ 9370 | 8 months, 4 and 7 years | Cognitive impairment | Bayley Scales of Infant Development, Stanford– Binet IQ (SBIQ), Wechsler Intelligence Scales for Children (WISC) and Wide-Range Achievement Tests (WRAT) |
| J. L. Richards, (2016) | Cohort | United States of America | 1450/ 1550 | 2, 5-6 years | Cognitive impairment, academic performance | Bayley Short Form-Research Edition (BSF-R) Mental Scale, Reading and mathematics achievement at kindergarten age: ECLS-B designed assessments |
| K. Baumgartel, (2020) | Cohort | Australia | 353/ 1152 | 12-16 months | Cognitive impairment | Ages and Stages Questionnaire (ASQ) |
| K. G. Noble, (2012) | Registry | United States of America | 35549/ 92501 | 8-9 Years | Academic Impairment | California Testing Bureau (CTB) Achievement Test |
| K. Stene-Larsen, (2014) | Cohort | Norway | 7109/ 30641 | 18 and 36 months | Cognitive impairment | Ages and Stages Questionnaire (ASQ) |
| L. D. Reid (2019) | Cohort | United States of America | 10964/ 22424 | 5-6 years | School readiness | kindergarten teacher assessment adapted from the work sampling system (WSS) |
| L. G. Smithers (2015) | Registry | Australia | 3374/ 4664 | 5 years | Cognitive impairment | Australian Early Development Index (AEDI) |
| L. M. Reyes (2019) | Cohort | Germany | 199/ 591 | 6 years | Social development | children’s social inhibition in an unfamiliar setting was assessed with a standardized experimental procedure of the child’s interaction with an adult stranger |
| M. A. Quigley, (2012) | Cohort | United Kingdom | 1596/ 5407 | 5 years | Cognitive impairment | foundation stage profile (FSP) |
| M. de Jong, (2018) | Cohort | Netherlands | 14/ 38 | 18 and 24 months | Cognitive impairment | The Utrecht Tasks of Attention in Toddlers using Eye tracking  (UTATE); Parental Attention Directing observation system (PAD); Bayley-III-NL |
| M. Hanly (2017) | Cohort | Australia | 20951/ 53445 | 5-6 years | Cognitive impairment | AEDC, formerly Australian Early Development Index (AEDI) |
| M. Hosozawa, (2021) | Cohort | United Kingdom | 3232/ 11286 | 3, 5, 7, 11 and 14 years | Social Development | Strengths and Difficulties Questionnaire (SDQ) |
| M. J. Berry (2018) | Registry | New Zealand | 131949/ 329280 | 4, 15 years | Social development, academic performance | Before School Checks (B4SCs), National Certificate of Educational Achievement (NCEA) |
| M. Wu, (2021) | Cohort | China | 727/ 1678 | 2 years | Cognitive impairment | Bayley Scales of Infant Development (BSID), raw scores were calculated and converted to index scores of MDI and PDI |
| M. X. Liu, (2023) | Cohort | China | 542/ 975 | Between 3 - 10 years | Motor impairment | standardized assessment for developmental coordination disorder (DCD), Movement Assessment Battery for Children-second edition (MABC-2) |
| N. Alterman, (2022) | Cohort | United Kingdom | 1498/ 5064 | 11 and 16 years | Academic performance | Academic achievement at Key Stage 2 and Key Stage 4 |
| N. Alterman, (2021) | Cohort | United Kingdom | 2460/ 3438 | 11 years | Special educational needs | Special educational needs (SEN) at any point (parent report, statement of SEN issued by local authority) |
| N. M. Talge, (2016) | Cohort | United States of America | 189/ 343 | 3-9 years | ADHD | Conners’ Parent Rating Scales Revise-Short Form: Revised (CPRS-RS) |
| N. Z. Rabie, (2015) | Registry | United States of America | 11527/ 24005 | 3-5 years | ADHD/ Developmental speech or language disorders | International Classification of Diseases, Clinical Modification, 9^th^ Revision (ICD-9-CM) Codes |
| P. E. Shah (2016a) | Cohort | United States of America | 1400/ 2350 | 5-6 years | School readiness | specialized battery of tests developed for the ECLS-B to assess  early reading and math skills |
| P. Shah, (2016b) | Cohort | United States of America | 1800/ 3200 | 9m, 24m,  3-4 years,  5-6 years | Cognitive impairment, school readiness | Bayley Short Form–Research Edition (BSF-R) Mental T scores, specialized reading and math assessments developed for the ECLS- B |
| T. M. Nielsen, (2021) | Cohort | Denmark | 288/ 286 | 6-8 years | Psychiatric disorders | parent version of the Strengths and Difficulties Questionnaire (SDQ) |
| Y. Xia, (2021) | Registry | Denmark | 388 416/ 1 198 605 | N/A | Neuropsychiatric disorders | International Statistical Classification of Diseases and Related Health Problems, Tenth Revision (ICD-10) codes |
| Z. Chen, (2022) | Cohort | China | 1288/ 2800 | 0-6 years | Cognitive impairment | Gesell Developmental Scale (GDS) |
| E. Yangin,  (2023) | Cohort | Turkey | 109/ 109 | 18-24 months | Cognitive impairment | Mental development index (MDI) and physical development index (PDI) |
| J. P. Bentley, (2016) | Registry | Australia | 34 465/ 44 832 | 4-6 years | Physical, Cognitive impairment and social development | AEDC, originally the Australian Early Development Index (AEDI) |
| E. Roe,  (2022) | Cohort | Australia | 631/ 1570 | 12, 24 and 36 months of age | Cognitive impairment | Ages and Stages Questionnaire (ASQ) |
| I. M. Zambrana,  (2015) | Cohort | Norway | 3507/ 18 029 | 18 and 36 months | Language scores | Maternal reported items derived from several established scales: the Norwegian version of the Ages and Stages Questionnaires, the modified Checklist for Autism in Toddlers, the Non-Verbal Communication Checklist, the Early Screening of Autistic Traits Questionnaire, the Social communication Questionnaire and a question about the complexity and  comprehensibility of child utterances |
| R. J. Burger, (2023) | Registry | Netherlands | 442 566/ 132 596 | 13+ years | Academic performance | School performance |
| N. Libuy,  (2023) | Registry | United Kingdom | 57 956/ 88 441 | 7, 11 years | Academic performance, special educational needs | Key Stage 1 and Key Stage 2 |
| D. Syrengelas, (2022) | Cohort | Greece | 559/ 528 | 0-19 months | Motor impairment | Alberta Infant Motor Scale (AIMS) |
| O. Rose,  (2013) | Cohort | Chile | 305/ 604 | 12 months | Cognitive impairment | Bayley Scales of Infant Developmen (BSID), Mental development index (MDI), physical development index (PDI) |
| R. Wiingreen, (2018) | Registry | Denmark | 56 180/ 114 972 | 6-18 years | Academic performance, Special educational needs | School difficulties were investigated by two different measurements 1) Special educational support in compulsory school and 2) Failing to complete compulsory school |
| E. V. Espel, (2014) | Cohort | United States of America | 70/ 66 | 3, 6, and 12 months | Social Development | Bayley Scales of Infant Development-II (BSID-II) |
| G. L. Wehby (2023) | Cohort | United States of America | 236 019/ 1 269 496 | 7-17 years | Academic performance | scores in national percentile rankings (NPRs)  on standardized school tests in math and reading |
| K. Haneda, (2024) | Cohort | Japan | 5 327/ 15 479 | 1 year | Cognitive impairment | Ages and Stages questionnaire (ASQ) |
| K. Hirata (2024) | Cohort | Japan | 21 533/ 44 075 | 3 years | Cognitive impairment | Ages and Stages questionnaire (ASQ) |
| C. Copper, (2023) | Cohort | United Kingdom | 468/ 1 774 | 11 years | Academic performance | standardized teacher assessments and national tests |
| S. Alenius, (2023) | Cohort | Finland | 39 751/ 164 474 | 7-16 years | Special educational needs | Registry data about mainstream or special education |
