# Supplementary material for: Cognitive and academic outcomes of large‐for‐gestational‐age babies born at early term: A systematic review and meta‐analysis
Source: Acta Obstet Gynecol Scand. 2024 Oct 30;104(2):288–301. doi: 10.1111/aogs.15001 (PMC11782071; doi:10.1111/aogs.15001)
Supplement: Supplementary file 9 — Table S4. [file AOGS-104-288-s001.docx]

Table S4 Three main primary outcomes and their definitions

| Primary outcome:  Cognitive scores (continuous cognitive standardized assessment scores) | | | |
| --- | --- | --- | --- |
| Study | Assessment | Additional description from study text | Age at follow-up |
| O. Rose (2013) | Bayley Scales of Infant and Toddler Development (BSID).-Mental Developmental Index (MDI) | The test has a standardized mean of 100 and SD of 15, based on a representative US sample of 1409 infants. the Mental Developmental Subscale showing moderate correlations with IQ at later ages. | 1-3 years |
| E.V. Espel (2014) | Bayley Scales of Infant and Toddler Development (BSID).-Mental Developmental Index (MDI) | Examiners were trained by a clinician with over 15 years of experience with the BSID and were directly supervised by a clinical psychologist. | 1-3 years |
| J. Hua (2019) | Bayley Scales of Infant and Toddler Development (BSID).-Mental Developmental Index (MDI) | We first obtained formal permission to translate and validate the BSID-III from the American publishers of this tool (Pearson). We then started developing a Chinese version of BSID-III, following the recommendations of Hambleton and Patsula and Herdman, Fox-Rushby and Badia for the translation and adaptation of a test,  taking into consideration conceptual, item, semantic, operational, measurement and functional equivalences. Each step of this process was presented in the results section. The Chinese version of the BSID-III was translated by a native Chinese speaker and independent  professional who adapted the items into context and culture. Subsequently, the final Chinese version of the BSID-III was retranslated into English by two native English speakers who were blinded to the original version. | 1-3 years |
| J.L. Richards (2016) | Bayley Scales of Infant and Toddler Development (BSID).-Mental Developmental Index (MDI) | Our analyses used children’s scale scores, which represented the item response theory (IRT)-based estimate of raw score on the full BSID-II | 1-3 years |
| J. L. Beauregard (2018a) | Bayley Scales of Infant and Toddler Development (BSID).-Mental Developmental Index (MDI) | Because the dataset did not report normed scores, our primary analyses used children’s scale scores. We also standardized scale scores to a normal distribution (mean = 0, standard deviation (SD), 1) in order to interpret results in terms of standard deviation differences between groups. | 1-3 years |
| E. Yangin Ergon (2023) | Bayley Scales of Infant and Toddler Development (BSID).-Mental Developmental Index (MDI) | Bayley and neurologic examinations of the patients were performed by the same specialist at the Developmental and Behavioral Pediatrics Unit. | 1-3 years |
| P. Shah (2016b) | Bayley Scales of Infant and Toddler Development (BSID).-Mental Developmental Index (MDI) | Infant Development. Developmental outcomes at 9 and 24 months were ascertained with the Bayley Short Form–Research Edition (BSF-R) Mental T scores. | 1-3 years |
| A. R. Bischoff (2017) | Bayley Scales of Infant and Toddler Development (BSID).-Mental Developmental Index (MDI) | The evaluation was performed by experienced professionals within 4 months of the time point when the child reached 36 months. | 1-3 years |
| J. F. Paulson (2014) | Bayley Scales of Infant and Toddler Development (BSID).-Mental Developmental Index (MDI) | To allow for comparison across BSID and reading  assessments, all scores were standardized by conversion to percentiles prior to analysis. | 1-3 years |
| S. Yang (2010) | Wechsler Abbreviated Scale of Intelligence (WASI) | The WASI consists of 4 subtests of vocabulary, similarities, block designs, and matrices. The WASI was translated from English to Russian and back-translated to ensure comparability of the Russian version. It was administered by the polyclinic pediatricians after extensive training and follow-up monitoring by child psychologists and psychiatrists in Minsk, Belarus. | 6.5 years |
| M. M. Costantine (2021) | Wechsler Preschool and Primary Scale of Intelligence (WPPSI) full scale test | The test has a standardized mean of 100 and SD of 15. | 5 years |
| J. L. Gleason (2021) | Wechsler Intelligence Scale for Children (WISC) | All developmental assessments  were normalized, such that a score of 100 equates to average | 7 years |
| J. L. Beauregard (2018b), | British Ability Scales II (BAS II) | BAS II word reading and  pattern construction scales | 7 years |
| I. M. Zambrana (2015) | maternal reported language skills | Our language comprehension and language production outcomes are latent variables based on previous Confirmatory Factor Analyses (CFA) work showing very good model fits with high non-overlapping factor loadings. The language comprehension constructs at 18 months (five items) and 36 months (seven items) captured  understanding of or appropriate responses to the language  utterances of others, and were measurement invariant across time. The language production construct at 18 months was based on one item enquiring whether the  child could utter eight or more words, in addition to ‘mama’ and ‘dada’, and at 36 months this construct captured the complexity of child utterances and conversations (seven items). These were thus not the same across time. | 1.5 years |
| B. Yu (2017) | Cognitive performance | was assessed by a series of tests in three domain areas – verbal, spatial and numerical ability – each with a 40-point scale. Test scores were standardized with a mean of zero and SD of one | 13 years |
| M. Zhang (2020) | Average DQ in Gesell | The items of the Chinese version of GDS are grouped into five neurodevelopmental domains: gross motor, fine motor, adaptability, language and social behavior. Maturity age can be used to calculate a development quotient (DQ), which is the maturity age divided by the chronological age, multiplied by 100. DQ is corrected if the infant is preterm. | 6 months |
| Primary outcome:  Cognitive impairment (Dichotomous categories included diverse indices) | | | |
| J. P. Bentley (2016) | Australian Early Development Census development Hazard | The primary study outcome was children who were Developmental Hazard Ratio, defined as being Developmental Vulnerable on ≥2 of the 5 main domains. | 4-6y |
| J. L. Gleason （2021） | WISC-full scale IQ--below average | All developmental assessments were normalized, such that a score of 100 equates to average. Children who scored <85 (one standard deviation below the mean) on any of the 4- or 7-year assessments  were classified as having below-average intelligence or achievement. | 7y |
| L.G. Smithers (2015), | Vulnerable on >=1 Australian Early Development Index (AEDI) domain | The AEDI is a holistic measure of children’s development at school entry (median age 5 years) that demonstrates predictive validity for later school achievement. In 2009, the AEDI was completed by teachers during a national census of children attending their first year of school. The AEDI includes 95 questions resulting in scores ranging from 0 to 10 across the following five developmental domains: physical  health and wellbeing, language and cognitive skills, emotional maturity, social competence, and communication and general knowledge. According to national reporting practices, responses are adjusted for age (in years) at entry to school and children in the lowest 10% of the population distribution of AEDI scores are considered developmentally  vulnerable on each domain. Children were also categorised according to whether they were vulnerable on one or more of the five domains. | 5y |
| G.K. Dhamrait (2021) | Vulnerable on >=1 Australian Early Development Index (AEDI) domain | The Australian Early Development Census (AEDC) is a national census of early childhood development spanning across five developmental domains: (i) Physical Health and Wellbeing, (ii) Social Competence, (iii) Emotional Maturity, (iv) Language and Cognitive Skills (school-based), and (v) Communication Skills and General Knowledge. The AEDC, originally the Australian Early Development Index (AEDI) is an adapted version of the Canadian Early Development Instrument (EDI). It is a teacher-completed instrument collected for all children in their first  year of full-time school (known as pre-primary in Western Australia, the year level prior to grade 1). The AEDC is conducted every three years, and results are reported at the national, state and territory, community, and local community levels. AEDC cut-off scores are based on the 2009 data collection, and apply to all subsequent AEDC data collections. Children who score < 10^th^ percentile in a given domain are classified as ‘developmentally vulnerable’ | 5y |
| E.Roe (2022), | Any Developmental delay using Ages and Stages Questionnaire (ASQ) | The ASQ comprises five domains: gross motor, fine motor, communication, adaptive and personal-social skills. Cut-off values to classify infants (1) in need of monitoring or continued surveillance, or  (2) who were at risk and needed referral to early intervention services were derived for each domain from the ASQ manual | 1-3y |
| H. K. Brown (2014), | Any Developmental delay using the Motor and Social Development Scale | Developmental delay was measured at 2 to 3 years using the Motor and Social Development Scale, which was developed by the US National Center for Health Statistics.30 The parent responds to 15  yes/no task performance questions which vary depending on the child’s  age), and the “yes” responses are summed.  Scores were standardized by 1-month age groups (M = 100, SD = 15),  and children scoring >=1 SD below the age-standardized mean were classified as having a delay. | 1-3y |
| K.Baumgartel (2020) | Any Developmental delay using Ages and Stages Questionnaire (ASQ) | Scores were then compared to established cut-points for each domain, with an abnormal score being defined as 2 standard deviations below the mean indicating clinically significant delay [25]. Infants with an abnormal domain score were classified as having developmental delay for that domain. | 1-3y |
| M. G. Eide (2009) | Intellectual Performance Score <= 3/9 (only men), | Intellectual performance was measured by a 53-min standardized  group intelligence test, which was developed in 1953 for the Norwegian  draft board and revised in 1962. The test included time-limited subtests organized by increasing difficulty covering three categories of items: verbal analogues, number series (calculation) and geometric figures (an abbreviated version of Raven’s Progressive Matrices) (31), comprising a total of 120 questions. All conscripts received standard instructions before the tests. Scores (with values from 1 to 9) are based on the normal distribution in 1962 with a mean of 5.0 and SD of 1.96. A low score was defined as <=3, corresponding to the 16th percentile of the distribution. | 18y |
| A. Fitzpatrick (2016), | the British Ability Scales Edition II Verbal Similarities test and the Cambridge  Neuropsychological Test Automated Battery Spatial Working Memory  test. | Z scores were dichotomized at 1 SD below the mean to indicate where a child was delayed on a test. 9, 26, 27 Z scores were reverse coded so that poorer performance was reflected in lower standardized  scores. | 11y |
| J Hua (2022) | Any Developmental delay using Ages and Stages Questionnaire (ASQ) | According to the cutoff provided by the ASQ-3 user’s guide, (39) a score that is more than two standard deviations below the mean indicates Suspected Developmental Delay (SDD) in each domain. | 4y |
| G. Poulsen (2013) | British Ability Scale <-1SD (average) | The BAS II is a battery of individually administered tests of cognitive and educational ability, suitable for use from ages 2 years and 6 months to 17 years and 11 months. The individual subscales are meaningful and can be analysed separately. Data were available on  the BAS II Naming Vocabulary Subscale (age 3 and 5 years) which measures vocabulary and expressive reasoning, the Picture Similarities Subscale (age 5 years) measures non-verbal reasoning, the Pattern Construction (age 5 and 7 years) measures spatial  abilities and the Word Reading subscale (age 7 years) involves verbal reasoning. | 7y |
| K. Stene-Larsen (2014) | language impairments/delay | Child communication impairments at age 36 months were assessed using 6 items from the ASQ measuring expressive (3 items) and  receptive (3 items) communication skills. To identify the children at risk  for clinically significant communication impairments, we set a cutoff of 2 SD above the cohort mean. | 3y |
| L.M. Reyes (2019) | social impairments | At age 6 years, children’s social inhibition in an unfamiliar setting  was assessed with a standardized experimental procedure of the child’s interaction with an adult stranger Based on the distribution of responses for the healthy (i.e., not neonatally hospitalized) full-term children (Figures 1 and 2), latencies of all children’s nonverbal  and verbal responses to the stranger were coded into 3 social approach categories: 1 = disinhibited (response nonverbal: <180 s; verbal: <180 s), 2 = normally responsive (response nonverbal: 180–188 s; verbal: 180–227 s) and 3 = inhibited (response nonverbal: >188 s, verbal >227). This response pattern corresponded with the experimental stimulus (i.e., the stranger inviting the child to play  after 180 s); thus, children were coded as not socially inhibited or disinhibited if they showed the expected developmentally-appropriate response to the social cue. | 6y |
| M.A. Quigley (2012) | not reached a good level of overall achievement in the foundation stage profile scales | The foundation stage profile (FSP) records the child’s achievement  as measured by their teacher at the end of their first school year, ‘foundation stage’. Teachers are trained in how to conduct the assessments, which are based on observations during the whole year. The FSP captures the ‘Early Learning Goals’ as a set of 13 assessment scales across six areas of learning (details in table 2).14 15 For each scale, the teacher gives the child 1–9 points according to the child’s progress in achieving the learning goals. Children achieving a scale score of ≥6 points are classified as working securely within the Early Learning Goals and are classified as having achieved a good level of development. Children who achieve a score of ≥78 points  across the 13 assessment scales (ie, an average of 6 points per  scale) and a score of ≥6 in each of the three ‘personal, social and emotional development’ scales and the four ‘communication,  language and literacy’ scales are classified as reaching a good level of overall achievement. | 5y |
| M. Wu (2021) | MDI delay (<85) | Lower MDI scores reflect poorer performance. To avoid potential underestimation of neurodevelopmental delay, neurodevelopmental delay was defined as 1 SD or more below the mean of the scores of the children in this study | 2y |
| J.J. Liang (2020), | Gesell developmental delay | Developmental delay was defined as any Gesell score <85 | 0-6y |
| Z. Chen (2022) | Gesell developmental delay | The score in each domain is expressed by developmental quotient (DQ), calculated as below: children’s estimated developmental age/ chronologic age× 100. According to the Chinese norm, the neurodevelopmental delay of each domain was defined as the DQ score below 85 | 0-6m |
| M.X. Liu (2023) | Cognitive delay (>=15^th^ percentile) | The age band 1 (3–6 years) and age band 2 (7–10 years)  of a standardized assessment for developmental coordination disorder (DCD), Movement Assessment Battery for Children-second edition (MABC-2) were used to test children’s motor impairment in the study. A standard total test score and standard scores of the three subtests (manual dexterity, aiming and catching, and balance) of the MABC-2 can be obtained based on the Chinese local norm. These scores  were then grouped as suspected DCD (at or below the 5^th^ percentile of the total test score), at risk of DCD (between the 6th and 16th percentiles of the total test score) and typical performance (above the 16th percentile of the total test score), according to the MABC-2 manual. | 3-10y |
| N. Z. Rabie (2015) | developmental speech or language disorders | Outcome measures were derived from Medicaid claims and were based on the presence of at least one ICD-9-CM code for the evaluated specific conditions: developmental speech or language disorders (315.3 to 315.39). | 3-5y |
| A. Adanikin (2022), | Developmental concern by parents report | At the end of a child health review, the health visitor uses the relevant Child Health Surveillance Programme–Preschool (CHSP-PS) review form to record their overall assessment of development in each domain as either “no concern” or “concern.” The categorization of assessment result for each domain is determined by overall responses—including the opportunity to observe or examine for key skills, and the ASQ score cutoff for respective domain | 2-3.5y |
| A. Z. Khambalia(2017) | Vulnerable on >=1 Australian Early Development Index (AEDI) domain | Developmental outcomes (at ages 4–7 years) derived from the AEDC identified children as developmentally vulnerable if they were <10th percentile in one domain and developmentally high risk if they were <10th percentile in two or more domains. | 4-7y |
| M. Hanly (2017), | Vulnerable on >=1 Australian Early Development Index (AEDI) domain | Children were classified as developmentally vulnerable if they scored in  the lowest decile on one or more of the five AEDC domains, based on the 2009 threshold | 5y |
| C. E. Frank (2018) | Average verbal ability | Verbal ability was assessed using the Revised Peabody Picture Vocabulary Test (PPVT-R), which was administered to NLSCY subjects through an in-person assessment. Children scoring less than or equal to the 15th percentile on the age-standardized score were classified as having poor verbal ability | 4-5y |
| M. M. Costantine (2021) | IQ<85 | Our primary outcome was the same as the original trial primary paper—child IQ at 5 years of age assessed using the Wechsler Preschool and Primary Scale of Intelligence – Third Edition (WPPSI-III) Full Scale test. | 5y |
| M. Zhang (2020) | IQ <85 | Gesell developmental tests were assessed at the Developmental Neuropsychology Laboratory and at age 1–6 months by four trained pediatricians, who were ‘blind’ to the prenatal and perinatal background of the infants. | 1-6m |
| K. Tamai (2020) | Unable to reach age-appropriate developmental milestones (average) | We assessed the impact of birth weight categories  on neurodevelopment status using behavioral development  at both of these ages. In the survey, the parents were  asked to provide a “yes” or “no” response according to  whether children had reached various age-appropriate motor,  language, and behavioral milestones (unable to listen carefully, unable to focus on one task, unable to remain patient, unable to express emotions, unable to act in group, unable to keep promise). | 5.5y |
| K. Haneda, M. Hosoya, K. Fujimori, S. Yasumura, H. Nishigori, M. Kuse, H. Kyozuka, H. Maeda, A. Sato, Y. Ogata and K. Hashimoto (2024) | Any Developmental delay using Ages and Stages Questionnaire (ASQ-3) | The ASQ-3 is a screening system conducted via a questionnaire answered by an infant’s guardian. The points scored in  each development category are summed, and evaluation  as to whether they surpass the cutof values is performed. We use cut of values of the Japanese translation of the ASQ-3. Values below the cutof were deemed as “clinical” neural development abnormalities | 1y |
| K. Hirata, K. Ueda, K. Wada, S. Ikehara, K. Tanigawa, T. Kimura, K. Ozono, T. Sobue, H. Iso and the Japan Environment and Children’s Study Group (2024) | Any Developmental delay using Ages and Stages Questionnaire (ASQ-3) | The ASQ-3 contained 30 items divided into 5 developmental domains (6 items per domain) as follows: communication, gross motor skills, fine motor skills, problem solving and personal-social. According to the guidelines of the ASQ-3, a score of >2.0 SD below the mean is the referral cut-off and indicates a need for further assessment. The ASQ-3 manual recommends that a child be considered as screen positive if his/her score falls below the referral cut-off in any one of the five  domains. | 3y |
| G. Jee, S. J. Kotecha, M. Chakraborty, S. Kotecha and D. Odd (2023) | Speech problems | The primary outcome was parent-reported speech problems in early childhood adjusted for clinical and demographic confounders in SGA and LGA infants compared with AGA infants. |  |
| Y. S. Chang, L. W. Chen, T. Yu, S. H. Lin and P. L. Kuo (2023) | Intellectual disability | Diagnosis-related variables were coded using the International Classification of Diseases, 9th Revision, Clinical Modification (ICD-9-CM). ID was ascertained using the codes 317-319. We included  individuals of the diagnosis of ASD or ID if they had at least one inpatient record or at least two outpatient records. For ID, we included only those who were diagnosed after age five (when the standardized intelligence quotient evaluation can be better performed) |  |
| Primary outcome:  Low academic performance | | | |
| A.K. Searle (2017), | low math, spelling, or reading scores | NAPLAN includes reading, writing, spelling, grammar/punctuation  and numeracy domains, with items reflecting Australian curricula.19 Scores on each domain (range 0–1000) are categorized into 10 bands. Grade 3 children generally fall in band 4 (scores around the high 300s/low 400s, SDs ∼60–90);19 those scoring in band 1 are considered ‘below national minimum standard (NMS)’, those in band 2 ‘at NMS’ and those in bands 3–10 ‘above NMS’. Children scoring at NMS typically demonstrate some basic elements of literacy/numeracy required at their grade level, but many require additional support.19 Our study outcome was scoring at/below NMS. | Grade 3 (8-9y) |
| J. L. Gleason (2021), | low math, spelling, or reading scores | WRAT Reading, Spelling, and Arithmetic as primary Outcomes, Children who scored <85 (one standard deviation below the mean) on any of the 4- or 7-year assessments were classified as having below-average intelligence or achievement. | 7 y |
| I. Kirkegaard (2006), | low math, spelling, or reading scores (learning difficulties) | The children’s teachers were then asked to compare the child with a typical child of the same age. The teachers classified the child according to whether he/she performed reading, spelling, and arithmetic: (1) some above average,(2) considerably above average, (3) on average, (4) some below average, or (5) considerably below average. They were also asked whether the child had received or was  presently being specially tutored and the type of tutoring, if any. Children with serious problems, that is, those who were assessed as being considerably below average at a particular skill and who received or had received special tutoring, were classified as having a learning disability. | 9-11y |
| P. Shah (2016a), | Poor school readiness | The reading assessment was formulated from existing instruments including the Peabody Picture Vocabulary Test, 3rd Edition, and Preschool Comprehensive Test of Phonological and Print Processing and measured markers of early literacy including basic reading skills (letter and word recognition, understanding letter-sound relationships, phonological awareness, sight word recognition, and understanding words in the context of simple sentences). The reliability of the early reading assessment is described by the item response theory (IRT) reliability coefficient, reported as 0.92 at kindergarten. Scores provide ability estimates in a particular domain and were reported as normally distributed theta scores which demonstrated a range of −2.11 to 3.09 (mean= 0.33, SD= 0.86) at kindergarten(22). The ECLS-B mathematics assessment incorporated items to test the following content areas: number sense, geometry, counting numerical operations, and pattern recognition. The item response theory reliability coefficient for the early mathematics assessment was also 0.92 at kindergarten. The mathematics theta scores demonstrated a range of −2.42 to 3.12 (mean= 0.38, SD= 0.80) at kindergarten(22). Because a performance of 1 or 1.5 standard deviations below the mean for age on standardized, norm-referenced tests in a specific area of development has been used previously as a threshold to identify children in need of educational services(24, 25), we defined “poor school readiness” by kindergarten reading and math theta scores falling ≥1.5 standard deviations below the mean. | 3y |
| G. Poulsen (2013) | low math scores (<-1SD) | The Numeracy Skills test (age 7 years) is developed from the Progress in Mathematics test which is used to test school children’s performance  in the UK and contains a set of arithmetical problems. | 7y |
| A. Z. Khambalia(2017) | low math, spelling, or reading scores | Educational outcomes assessed in year 3 (at ages 7–9 years)included reading and numeracy, the components of NAPLANassessment that are the most consistent over time. Reading andnumeracy scores were categorised as less than one standard deviation below the mean (low score), more than one standard deviationabove the mean (high score) and average (remaining values in between low and high scores). Children who were exempt from sitting the test due to complex or severe disability were classified as having a low score. The proportion of children below the NAPLAN national minimum standard in reading and numeracy are also reported.19 For each test, scores are nationally  equated across grades and years to ensure that results are comparable between grades and over time. | 7-9y |
| D. F. MacKay (2010), | special educational needs | The Department of Education defines SEN as a learning difficulty that requires special educational provision. SEN includes both children with learning disabilities (including dyslexia, dyspraxia, autism, Asperger’s syndrome, and attention deficit hyperactivity disorder), as well as children with physical disabilities that impact on learning (including some children with hearing, motor, and visual impairments). We used data from the 2005 school census. The school census is undertaken annually in September and the data are provided by head-teachers at each school and collated by their local education authority. The school  census covers all schools in Scotland irrespective of their funding  source and includes local authority, grant-aided, independent,  and self-governing schools. The response rate is 99.8%. It includes all primary and secondary school children. | 4-19y |
| N. Libuy (2023), | special educational needs | We also evaluated the percentage of children who ever had SEN provision in primary school (defined as those with a statement of SEN or an Education Health & Care Plan or Action, Action Plus or Support) between the academic year 2010/11 (when our cohort were  in reception class age 5) and 2015/16 (Year 6, age 11). | 5y, 11y |
| R. Wiingreen (2018), | special educational needs | Special educational support: In Denmark children with school difficulties can receive special educational support in compulsory school either as supportive measures in the general class or in a special class. Based on a pedagogical and psychological assessment the headmaster of the school, in consultation with the parents and the pupil, decides whether the pupil must receive special education [18]. It is mandatory by law that the school administration reports data on special educational support to the individual pupil to the national Danish special educational register once a year. Children receiving at least 9 hours special educational support weekly are registered. For the present analyses data from the special educational register were  collected and special educational support was defined if a pupil was registered with special education and thereby received at least 9 hours support weekly. | 3-18y |
| M. J. Berry (2018) | special educational needs | Children are required to be enrolled in primary school in New Zealand by age 6 years. These data exclude the 0.8% of children in New Zealand who are homeschooled.‍46 Children with additional learning needs receive support through the Resource Teachers: Learning and Behaviour (RTLB) service. The Reading Recovery program is an early literacy intervention used to target children who are falling behind after 1 year at school.‍47 We calculated the rates of primary school, RTLB service, and Reading Recovery enrollment for each gestational week group. | 6y |
| R. J. Burger（2023） | lower secondary school level | The outcome of interest was offspring school performance measured on a standardized school performance test and secondary  school level reached at the end of primary school at age 12. The Dutch education system differs from that in many other countries in that at the end of primary school, around 12 years of age, children are divided over four different levels of secondary education according to their intellectual ability. All children in the last year of regular primary education in the Netherlands are obliged to take a test officially recognized by the Dutch government to guide the choice of level of secondary education. Children in special education are not obliged to take the standardized test. The most common test is the Central Final Test (formerly known as Cito Test), taken by 50%–65% of children. It covers language, arithmetic/mathematics and study skills. The school performance score ranges from 501 to 550 with a mean of 535. A score of 501–536 equals pre-vocational secondary school level (in this paper  referred to as ‘lower’); a score of 537 or higher matches senior  general or pre-university (in this paper referred to as ‘higher’) secondary school level. | 12y |
| K. Lindstroem (2007) | lower total education years | Education was categorized as “basic” if the study participant had completed no more than the compulsory 9 years of primary school, and as “postsecondary” if >=1 educational level had been completed  after secondary school. | 23-29y |
| A. Hedges (2021) | Lower than average in academic skills | When the child was 9 years old, their teacher was asked: “Overall,  how would you rate this child’s academic skills in each of the  following areas, compared to other children of the same grade level?”  The areas were mathematics, science and social studies, and language and literacy, and the response choices for each were far  below average, below average, average, above average, or far above  average. For each area, we created a binary outcome for average, above average, or far above average (versus far below average or below average) to characterize the child not being behind in terms of performance, but we assessed sensitivity to other cutoffs and to continuous measures. Additionally, we created a binary outcome for average, above average, or far above average ratings in all 3 subjects. | 9y |
| N. Alterman (2022) | not achieving the expected level of attainment at Key Stage 2 | At the end of primary school, when children are 11 years of age, pupils’ attainment at KS2 is assessed using standardised tests in English and Mathematics, alongside a teacher’s assessment of their schoolwork in these subjects. Our study’s main outcome measure for KS2 was not achieving the expected level of attainment (Level 4 or above) in  both English and Mathematics | 16y |
| E Chan (2014) | Not achieved level 2 in Key Stage 1 | In England, all state-funded and some private schools progress through the national curriculum comprising of four ‘key stages’  which begin in year 1 (age 5–6 years) and are completed by the  end of year 11.17 Key Stage 1 (KS1) covers coursework completed  between ages 5–7 years in five key domains: reading, writing, speaking and listening, mathematics and science. The KS1 statutory assessments comprise teacher evaluations of the student’s academic achievement throughout the school year in each domain according to uniform criteria, aided by standardized KS1 tests in most subject areas.18 At KS1, children generally perform between level 1 (below expected level) to level 3 (considerably above the expected level), with adequate performance categorized as achieving level 2 or above.  KS1 results were converted into binary outcomes with adequate  performance defined as level 2 or 3, and below the expected  level as level 1 or below. The proportion of children performing  below expected levels for each outcome was compared in each  gestational age group against the full-term reference group.  Children who attained level 2 or above in reading, writing and  mathematics were categorised as achieving adequate general  school performance, and the primary outcome was defined as  not having reached this level. | 5-7y |
| B. Yu (2017) | Not attained higher education | The outcome of whether the subject attained higher education  or not was assessed at the age of 48. Those who had  postsecondary or a higher level of education were classified  as having attained higher education. | 48y |
| K. O. Duffany (2020) | Low school test score (average) | Outcomes assessed included children meeting proficiency on the third grade standards-based mathematics and English language arts assessments and being referred for special education at any time. Nationally in the US, statewide educational assessments are required as part of the No Child Left Behind Act of 2001, Public Law 107–110, with the goal for all students to reach proficiency on each subject-based assessment. The development process for these subject-based standards assessments includes a rigorous process at the state-level including assessment of content and construct validity [18].  Student performance scores on these assessments are reported on a four-point scale. A score of 1 or 2 indicates the student did not meet proficiency; conversely, a score of 3 or 4 indicates the student met or exceeded proficiency. We assessed the bivariate outcome: met/did not meet proficiency on the mathematics assessment and met/did not meet proficiency on the English Language arts assessment. | 8-9y |
| L. G. Smithers (2019) | Low school test score (average) | School assessments were obtained from the National Assessment Program – Literacy and Numeracy (NAPLAN), which involves  assessments of reading, writing, spelling, grammar, and numeracy in grade three. Scores were dichotomized at performing above the national minimum standard or not. Children who perform at or below the standard typically require classroom learning support. | 8y |
| G. L. Wehby (2003) | Average school test score | Student scores on the standardized school tests for children in grades 2 through 11 and available through school year 2017 to 2018 obtained from the Iowa Testing Programs. The school tests are based on the Iowa Tests of Basic Skills, Iowa Tests of Educational  Development, and Iowa Assessments. Both public and private schools routinely administer these tests, and scores on these tests have been previously associated with conceptually relevant health and policy factors, further supporting their validity. This study focuses on math and reading tests, the 2 most commonly tested subjects. The 2 outcome measures are standardized test scores on math and reading, shown in national percentile rankings (NPRs), so the differences in scores across gestational ages can be directly interpreted as differences in percentile rankings relative to a national sample. | 7-17y |
| C. Copper, A. Waterman, Ch. Nicoletti, K. Pettinger, L. M. Sanders, L. J. B. Hill (2023) | Low school test score (average) | Binary variables of whether a child reached the ‘expected’ level  of overall educational achievement across subjects at the ages of 5, 6, 7 and 11 years. The achievement levels are measured using standardized teacher assessments and national tests. Primary education in England (age 4-11 years) divides into three stages (Early Years Foundation Stage [EYFS]; Key Stage 1 [KS1]; Key Stage 2 [KS2]). At the end of each stage statutory assessment occurs against national standards. | 11y |
| S. Alenius, E. Kajantie, R. Sund, M. Nurhonen, P. Haaramo, P. Näsänen-Gilmore, S. Lemola, K. Räikkönen, D. D. Schnitzlein, D. Wolke, M. Gissler and P. Hovi (2023) | Special education needs | The outcomes were: (1) the proportions of individuals in mainstream education, special education, and discontinued compulsory education (in subsequent analyses we included only students in mainstream education to ensure the comparability of the educational attainments). | 7-16y |
| A. Gustafsson, A. Bonnevier, K. Källén (2023) | Special education needs | The children's final school grades achieved at approximately 16 years of age were extracted from the Swedish School Grade Register, kept by Statistics Sweden. The register includes information on school achievements for all children enrolled in mainstream school since 1988. No register exists that contains information on children enrolled in special needs schools. Instead, children who had not died, and for whom no information on emigration or school grades from mainstream school was available, were assumed to have been educated in special needs schools. | 16y |
