# Supplementary material for: Cognitive and academic outcomes of large‐for‐gestational‐age babies born at early term: A systematic review and meta‐analysis
Source: Acta Obstet Gynecol Scand. 2024 Oct 30;104(2):288–301. doi: 10.1111/aogs.15001 (PMC11782071; doi:10.1111/aogs.15001)
Supplement: Supplementary file 11 — Table S6. [file AOGS-104-288-s002.docx]

Table S6 Sensitivity analyses of cognitive scores using crude or most-adjusted data

| **37w vs 40w** | **Crude data** | **Most-adjusted data** |
| --- | --- | --- |
| O. Rose (2013) | NA | 37w mean: 102.60; SD: 11.40  40w mean: 105.10; SD: 11.50 |
| S. Yang (2010) | Mean difference: -2.60; 95% CI: (-3.70, -1.40) | Mean difference: -1.70; 95% CI: (-2.70, -0.70) |
| E.V. Espel (2014) | NA | Mean difference: -1.99; 95% CI: (-3.51, -0.45) |
| J. Hua (2019) | Mean difference: -1.21; 95% CI: (-2.72, 0.29) | Mean difference: -2.26; 95% CI: (-4.28, -0.24) |
| J.L. Gleason (2021) | NA | Mean difference: -1.54; 95% CI: (-2.49, -0.59) |
| J.L. Richards (2016) | 37w mean: 125.50; SD:11.70  40w mean: 128.20; SD:10.50 | Mean difference: -0.80; 95% CI: (-1.90, 0.20) |
| Pooled standard mean difference | -0.22 (-0.29, -0.16) | -0.13 (-0.21, -0.05) |
|  |  |  |
| **38w vs 40w** | **Crude data** | **Most-adjusted data** |
| O. Rose (2013) | NA | 38w mean: 103.40; SD: 12.30  40w mean: 105.10; SD: 11.50 |
| S. Yang (2010) | Mean difference: -0.60; 95% CI: (-1.10, -0.01) | Mean difference: -0.40; 95% CI: (-1.10, 0.20) |
| E.V. Espel (2014) | NA | Mean difference: -1.99; 95% CI: (-3.51, -0.45) |
| J. Hua (2019) | Mean difference: 0.97; 95% CI: (0.06, 1.99) | Mean difference: 0.12; 95% CI: (-1.18, 1.42) |
| J.L. Gleason (2021) | NA | Mean difference: -0.83; 95% CI: (-1.54, -0.13) |
| J.L. Richards (2016) | 38w mean: 126.60; SD: 10.40  40w mean: 128.20; SD: 10.50 | Mean difference: -0.10; 95% CI: (-0.80, 0.70) |
| Pooled standard mean difference | -0.03 (-0.16, 0.09) | -0.04 (-0.08, 0.002) |
|  |  |  |
| **Early-term vs Full-term** | **Crude data** | **Most-adjusted data** |
| I.M. Zambrana (2015) | Language comprehension  mean difference: -0.23; 95% CI: (-0.32, -0.15)  Language production  mean difference: -0.19; 95% CI: (-0.26, -0.12) | Language comprehension  mean difference: -0.16; 95% CI: (-0.26, -0.07)  Language production  mean difference: -0.14; 95% CI: (-0.22, -0.06) |
| J.L. Beauregard (2018a) | NA | Mean difference: -0.20; 95% CI: (-0.80, 0.40) |
| J.L. Beauregard (2018b) | NA | Mean difference: -0.07; 95% CI: (-0.12, -0.02) |
| J.L. Richards (2016) | NA | Mean difference: -0.2; 95% CI: (-0.80, 0.4) |
| E. Yangin Ergon (2023) | Early-term mean: 85.35; SD: 10.99  Full-term mean: 89.97; SD: 14.14 | Early-term mean: 85.35; SD: 10.99  Full-term mean: 89.97; SD: 14.14 (only have crude data) |
| P. Shah (2016b) | NA | Early-term mean: 93.40; SD: 0.86  Full-term mean: 93.70; SD: 0.80 |
| Pooled standard mean difference | -0.22 (-0.40, -0.04) | -0.14 (-0.26, -0.02) |
|  |  |  |
| **LGA vs AGA** | **Crude data** | **Most-adjusted data** |
| A.R. Bischoff (2017) | LGA mean: 99.10; SD: 10.41  AGA mean: 98.47; SD: 10.43 | LGA mean: 99.10; SD: 10.41  AGA mean: 98.47; SD: 10.43  (only have crude data) |
| B. Yu (2017) | Mean difference: 0.10; 95% CI: (0.05, 0.16) | Mean difference: 0.057; 95% CI: (0.003, 0.107) |
| J.F. Paulson (2014) | NA | LGA mean: 99.10; SD: 10.41  AGA mean: 98.47; SD: 10.43 |
| M.M. Costantine (2021) | LGA mean: 93.60; SD: 13.70  AGA mean: 95.10; SD: 14.90 | LGA mean: 93.60; SD: 13.70  AGA mean: 95.10; SD: 14.90  (only have crude data) |
| M. Zhang (2020) | LGA mean: 95.64; SD: 23.34  AGA mean: 94.14; SD: 23.52 | LGA mean: 95.64; SD: 23.34  AGA mean: 94.14; SD: 23.52  (only have crude data) |
| Pooled standard mean difference | 0.07 (-0.02, 0.15) | 0.06 (0.01, 0.11) |

NA: not available; LGA: large-for-gestational-age; AGA: appropriate-for-gestational-age; CI: confidence interval; SD: standard deviation
