# Supplementary material for: Cognitive and academic outcomes of large‐for‐gestational‐age babies born at early term: A systematic review and meta‐analysis
Source: Acta Obstet Gynecol Scand. 2024 Oct 30;104(2):288–301. doi: 10.1111/aogs.15001 (PMC11782071; doi:10.1111/aogs.15001)
Supplement: Supplementary file 12 — Table S7. [file AOGS-104-288-s004.docx]

**Table S7**

GRADE summary table

| Outcomes | Exposures | No of Participants (studies) | Quality of the evidence (GRADE) | Pooled estimate (95% CI) |
| --- | --- | --- | --- | --- |
| Cognitive scores | Born at 37 weeks | 27912  (6 studies) | **LOW^1,3,4^**  upgraded because of dose-response gradient and downgraded due to publication bias | SMD -0.13 (-0.21, -0.05) |
|  | 38 weeks | 33004  (6 studies) | **MODERATE^1,3,4^**  upgraded because of dose-response gradient | SMD -0.04 (-0.08, 0.002) |
|  | early-term | 39171  (6 studies) | **VERY LOW^1.2,3^**  downgraded due to unexplained heterogeneity | SMD -0.14 (-0.26, -0.02) |
| Cognitive impairment | Born at 37 weeks | 92403  (4 studies) | **LOW^1,2,3,4^**  upgraded because of dose-response gradient and downgraded due to unexplained heterogeneity | RR 1.28 (1.13, 1.45) |
|  | 38 weeks | 120973  (4 studies) | **MODERATE^1,3,4^**  upgraded because of dose-response gradient | RR 1.12 (1.03, 1.21) |
|  | early-term | 1005005  (19 studies) | **VERY LOW^1,2,3^**  downgraded due to unexplained heterogeneity | RR 1.18 (1.12, 1.24) |
| Low academic performance | Born at 37 weeks | 576869  (5 studies) | **VERY LOW^1,2,3,4^**  upgraded because of dose-response gradient and downgraded due to publication bias, unexplained heterogeneity | RR 1.17 (1.02, 1.35) |
|  | 38 weeks | 614005  (6 studies) | **LOW^1,3,4^**  upgraded because of dose-response gradient and downgraded due to publication bias | RR 1.10 (1.01, 1.19) |
|  | early-term | 2921251 (15 studies) | **VERY LOW^1,3^**  downgraded due to publication bias | RR 1.14 (1.09, 1.20) |
| Average school test score | Born at 37 weeks | 1519913  (3 studies) | **LOW^1,3^** | SMD -0.05 (-0.11, 0.01) |
|  | 38 weeks | 1848425  (3 studies) | **LOW^1,3^** | SMD -0.02 (-0.06, 0.01) |
|  | early-term | 49508  (4 studies) | **LOW^1,3^** | SMD -0.04 (-0.06, -0.02) |
| Cognitive scores | LGA | 16774  (5 studies) | **MODERATE^1,3,5^**  upgraded because of plausible residual confounding | SMD 0.06 (0.01, 0.11) |
| Cognitive impairment | LGA | 1252667  (8 studies) | **MODERATE^1,3,5^**  upgraded because of plausible residual confounding | RR 0.94 (0.92, 0.97) |
| Low academic performance | LGA | 3034929  (7 studies) | **MODERATE^1,3,5^**  upgraded because of plausible residual confounding | RR 0.94 (0.90, 0.98) |
| * The basis for the assumed risk (e.g. unexplained heterogeneity) is provided in footnotes. The corresponding risk (and its 95% confidence interval) is based on the assumed risk and the pooled estimate of the exposure (and its 95% CI)  GRADE: Grading of Recommendations, Assessment, Development and Evaluation; CI: confidence interval; RR: risk ratio; SMD: standardized mean difference; LGA: large-for-gestational-age | | | | |
| GRADE Working Group grades of evidence  High quality: Further research is very unlikely to change our confidence in the estimate of effect.  Moderate quality: Further research is likely to have an important impact on our confidence in the estimate of effect and may change the estimate.  Low quality: Further research is very likely to have an important impact on our confidence in the estimate of effect and is likely to change the estimate.  Very low quality: We are very uncertain about the estimate. | | | | |
| ** The vast majority of studies included in our systematic review are observation studies and the quality of evidence was initially set as low.  ^1^  Majority of studies were assessed as low risk of bias, and only a few were assessed as relatively low risk of bias according to the Newcastle-Ottawa Scale. Therefore, we did not downgrade the quality of evidence due to the risk of bias as the overall risk of bias was felt to be very low.  ^2^  The heterogeneity was evaluated by I^2^ and Tau^2^. When I^2^ is large (>50%), but Tau^2^ is very small (i.e. <0.01), it suggests that large heterogeneity suggested by I^2^ was dependent on the large sample size. We did not downgrade the quality of evidence in this case. Overall, we decided to downgrade by one level when both test estimates were large.  ^3^  The optimal information size criterion was met (>2000 according to our sample size calculation) for all the outcomes. We used SMD of cognitive score>0.13 as a compelling rationale for a threshold as 0.13*15=1.95≈2 is the threshold of clinical significance of IQ difference. Overall, we decided not to rate down for imprecision as all 95% CI of either cognitive score SMD or adverse event odds ratio excludes no effect or exclude important benefit or harm.  ^4^ We used essentially the same individual studies to explore the effects of birth at 37 and 38 gestational weeks, respectively. As gestational weeks increased, cognitive abilities increased accordingly. It suggests a dose-response gradient. We decided to rate up by one level when other risks were unlikely to change the trends.  ^5^ Compared to children born at normal weight, LGA children are more likely to be treated as a disadvantaged group in studies and thus receive lower assessment scores. Because the plausible biases due to this residual confounding would diminish the demonstrated effect, we decided to upgrade the certainty by one level. | | | | |
